# The Douglas-Fir Genome Sequence Reveals Specialization of the Photosynthetic Apparatus in Pinaceae

**DOI:** 10.1534/g3.117.300078

**Published:** 2017-07-27

**Authors:** David B. Neale, Patrick E. McGuire, Nicholas C. Wheeler, Kristian A. Stevens, Marc W. Crepeau, Charis Cardeno, Aleksey V. Zimin, Daniela Puiu, Geo M. Pertea, U. Uzay Sezen, Claudio Casola, Tomasz E. Koralewski, Robin Paul, Daniel Gonzalez-Ibeas, Sumaira Zaman, Richard Cronn, Mark Yandell, Carson Holt, Charles H. Langley, James A. Yorke, Steven L. Salzberg, Jill L. Wegrzyn

**Affiliations:** * Department of Plant Sciences, University of California, Davis, California 95616; † Department of Evolution and Ecology, University of California, Davis, California 95616; ‡ Institute for Physical Sciences and Technology, University of Maryland, College Park, Maryland 20742; § Center for Computational Biology, McKusick-Nathans Institute of Genetic Medicine, Johns Hopkins University, Baltimore, Maryland 21205; ** Department of Ecology and Evolutionary Biology, University of Connecticut, Storrs, Connecticut 06269; †† Department of Ecosystem Science and Management, Texas A&M University, College Station, Texas 77843; ‡‡ Pacific Northwest Research Station, United States Forest Service, Corvallis, Oregon 97331; §§ Department of Human Genetics, University of Utah, Salt Lake City, Utah 84112; *** Department of Biomedical Engineering, Johns Hopkins University, Baltimore, Maryland 21218; ††† Department of Computer Science, Johns Hopkins University, Baltimore, Maryland 21218; ‡‡‡ Department of Biostatistics, Johns Hopkins University, Baltimore, Maryland 21205

**Keywords:** gymnosperm, mega-genome, conifer, genome assembly, shade tolerance, annotation

## Abstract

A reference genome sequence for *Pseudotsuga menziesii var. menziesii* (Mirb.) Franco (Coastal Douglas-fir) is reported, thus providing a reference sequence for a third genus of the family Pinaceae. The contiguity and quality of the genome assembly far exceeds that of other conifer reference genome sequences (contig N50 = 44,136 bp and scaffold N50 = 340,704 bp). Incremental improvements in sequencing and assembly technologies are in part responsible for the higher quality reference genome, but it may also be due to a slightly lower exact repeat content in Douglas-fir *vs.* pine and spruce. Comparative genome annotation with angiosperm species reveals gene-family expansion and contraction in Douglas-fir and other conifers which may account for some of the major morphological and physiological differences between the two major plant groups. Notable differences in the size of the NDH-complex gene family and genes underlying the functional basis of shade tolerance/intolerance were observed. This reference genome sequence not only provides an important resource for Douglas-fir breeders and geneticists but also sheds additional light on the evolutionary processes that have led to the divergence of modern angiosperms from the more ancient gymnosperms.

As recently as 2012, no full-genome sequences of gymnosperm representatives were available, but several conifer genome-sequencing projects were underway and results were eagerly anticipated for the expected insights into gymnosperm genome evolution and function and applications of that information for applied research (Mackay *et al.* 2012). Today, technological and bioinformatics advances have made feasible the sequencing of huge genomes, and reference genome sequences for four conifer species in two genera have been released: Norway spruce [*Picea abies* (L.) Karst, 19.6 Gbp; Nystedt *et al.* 2013], white spruce [*Picea glauca* (Moench.) Voss, 20.8 Gbp; Birol *et al.* 2013; Warren *et al.* 2015], loblolly pine (*Pinus taeda* L., 20.1 Gbp; Neale *et al.* 2014; Zimin *et al.* 2014), and sugar pine (*Pinus lambertiana* Dougl., 34.1 Gbp; Stevens *et al.* 2016). To this body of work, the draft genome sequence of a species in a third conifer genus is added, that of Douglas-fir, *Pseudotsuga menziesii* (Mirb.) Franco, reported here.

Douglas-fir is distributed widely across much of western North America, from British Columbia to Mexico (∼4500 km), from the Pacific coast to the eastern slopes of the Rocky Mountains, and from sea level to 3000 m elevation where it grows on over 20 million ha (Hermann and Lavender 1990; Smith *et al.* 2001). At least two varieties are recognized: *P. m*. var. *glauca* is found in the interior West and *P. m*. var. *menziesii* along the Pacific slopes. Across much of its range the species is considered a keystone species in large, critical ecosystems (Lipow *et al.* 2003). It is the most economically important timber species in western North America (Howe *et al.* 2006), and is widely planted abroad, in Europe, New Zealand, Australia, and Chile (Hermann and Lavender 1999).

*Pseudotsuga* is one of 11 genera within the Pinaceae family (Eckenwalder 2009; Farjon 2010), phylogenetically most closely related to *Larix* (Gernandt and Liston 1999). Douglas-fir is unique in the genus and the family in having a chromosome number of 2*n* = 26 (Doerksen and Ching 1972), in contrast to *Pseudolarix amabilis* (J. Nelson) Rehd. with 2*n* = 44 (Sax and Sax 1933) and the family’s other 200+ species, which have 2*n* = 24. The species is predominantly outcrossing and exhibits high levels of genetic variation for virtually all traits evaluated. Genetically, it is among the best studied of conifers, and the focus of both intensive and extensive breeding efforts around the world (reviewed in Howe *et al.* 2006). Comprehensive Douglas-fir genetic resources exist including provenance (Krakowski and Stoehr 2009; Isaac-Renton *et al.* 2014), genecological (St. Clair *et al.* 2005), and breeding and testing (Howe *et al.* 2006) populations. A wide array of allozyme and DNA markers (RFLPs, RAPDs, SSRs, and SNPs) have been developed and used routinely in seed orchard and breeding research, genetic mapping (Jermstad *et al.* 1998), QTL discovery (Jermstad *et al.* 2003), and association mapping (Eckert *et al.* 2009). Gene models have also been developed for Douglas-fir using RNA-Seq methods (Müller *et al.* 2012; Howe *et al.* 2013; Cronn *et al.* 2017), and variation in transcribed regions has been translated into SNP assays (Howe *et al.* 2013) that are being substantially improved with new genomic resources.

## Materials and Methods

### Tissue collection and DNA extraction

The source of seed and needles was tree 412-2, property of Weyerhaeuser Corporation. The seeds had been collected from tree 412-2 and maintained as Lot #79A002 in the germplasm collection at the Institute of Forest Genetics, US Forest Service, Placerville, CA. Needles were collected by Kim Arnold (Weyerhaeuser Company) on April 7, 2014. Megagametophyte tissue was dissected from seeds under a stereomicroscope and DNA was extracted using the Machery-Nagel NucleoSpin Plant II kit. DNA was extracted from nuclei isolated from needles using the methods described in Zimin *et al.* (2014).

### Construction of paired-end (long and short) libraries

A set of short-fragment libraries for deep paired-end sequencing was constructed from the haploid DNA of a single megagametophyte [see Zimin *et al.* (2014) for details in Methods section]. The DNA yield was 2.39 µg and this was fragmented by sonication, end-repaired, A-tailed, and ligated with standard Illumina forked adapters. Adapter-ligated DNA was size-selected by running it on a 2% agarose gel and cutting a series of narrow bands. The recovered DNA was amplified by 10 cycles of enrichment PCR with KAPA HiFi HotStart master mix, using barcoded primers and up to 10 ng of size-selected template per 50 µl reaction. The result was 26 barcoded fractions with insert sizes ranging from 238 to 710 bp (Supplemental Material, Table S1 in File S1).

Libraries for long-range linking were made using the Nextera Mate Pair Library Preparation Kit (Illumina) following the Gel-Plus protocol. The needle DNA was first treated with 0.33 µl of PreCR mix (New England Biolabs) per microgram of DNA. Bst DNA polymerase and ThermoPol buffer (New England Biolabs) were substituted for the kit-supplied strand-displacement enzyme and buffer in cases where kit volumes ran low. All size selections were performed using 0.6% MegaBase agarose (Bio-Rad) gels run overnight on a Bio-Rad FIGE-Mapper. For libraries with longer inserts, the ratio of tagmentation enzyme to DNA was reduced to 1 µl enzyme per microgram DNA to create a higher molecular weight distribution of fragments. In these cases, six tagmentation reactions were combined before size selection, and the size selection was repeated once to improve size specificity. In this way, 21 libraries were created from fragments with sizes ranging from ∼3.5 to ∼24 kb (Table S2 in File S1). All mate-pair sequences were subsequently processed through NextClip (Leggett *et al.* 2014). Reads not containing a recognized junction fragment (NextClip categories A, B, and C) were removed.

### Genome assembly

In total, the reads for this project represented ∼61× coverage (based on an estimated genome size of 16 Gbp) in paired-end sequences from short fragments, ranging from 250 to 635 bp in length, and 11× coverage in paired-end sequences from longer fragments, ranging from 3.3 to 24.8 Kbp. There were 3,718,542,883 pairs of reads from the short-fragment libraries and 700,151,824 pairs of reads from the long-fragment libraries. All reads were 151 bp long.

The assembly was produced by first error-correcting all Illumina reads using MaSuRCA 2.3.2 (Zimin *et al.* 2013), and then building contigs using the short-fragment data only using SOAPdenovo2 (Luo *et al.* 2012). These contigs were then used to “clean” the longer fragments, which were produced using a different paired-end library method (sometimes called a “mate-pair” protocol) that is prone to errors. This cleaning procedure removed PCR duplicates, in which >1 pair of reads is generated from the same fragment, and also “short innies,” in which two reads are inward-facing and close together rather than outward-facing and far apart. We were able to recognize “innies” by discovering that they had a possible alignment to the contigs built in the first round that placed them too close together.

Following these data-cleaning steps, SOAPdenovo2 (with the parameters “-K 99 -k 63”) was used to assemble both the long and short fragment-pairs, with the short pairs used for contigging and all pairs used for scaffolding. This produced an initial assembly containing 1,311,227 scaffolds ranging in length from 200 to 3,901,907 bp, with a total size of 14.77 Gbp. Considering all scaffolds and contigs down to 100 bp, the total length was 16.6 Gbp.

Next a series of gap-closing steps were run. First, the SOAPdenovo GapCloser was used to fill in gaps totaling 87,699,202 bp in length. Second, the MaSuRCA gap-closing module filled in many more gaps, totaling 377,845,540 bp. The reason for using two gap closers in this sequence is that SOAPdenovo2 and MaSuRCA gap closers employ different algorithms. The SOAPdenovo gap closer closes smaller gaps by looking for Illumina reads that span the gap and match exactly on both sides of the gap by at least *k* bases (we used *k* = 63). The MaSuRCA gap closer creates a mini-assembly of the gap first by creating a local bin of Illumina reads that map to 200 bp of sequence on each side of the gap and their mates, and then by looking for a unique path in at least one direction through a *k*-mer graph for 19 ≤ *k* ≤ 150 using *k*-mers from the reads in the bin that match exactly at least 200 bases on each side of the gap. Thus, the MaSuRCA gap closer is able to close gaps that are longer than a read, but shorter than the length of a fragment for Illumina paired-end reads. These two steps were followed by a procedure to remove short contigs and scaffolds that were contained within newly closed gaps and were therefore redundant.

### Annotation

Custom as well as existing methods for repeat and gene annotation and analysis were followed to contend with the large and complex conifer genome. Details of the processes are provided in the *Methods* section in File S1. The investigation details reported here are focused on the comparative gene-family analysis and the related shade-tolerant and shade-intolerant characters of the conifers.

### Genes involved in light-harvesting complexes, photoreception, and defense

For comparative analysis, conifer orthologs of six light-harvesting complex photosystem II proteins (LHCIIb), two reaction center proteins (D1/D2), proteins involved in photosystem state changes, the pH sensor protein (PsbS), thylakoid associated kinase (STN7), H subunit (PSAH1) of photosystem I (PSI), and Violaxanthin-deepoxidase (VDE1) were identified through protein BLAST search in NCBI and 1KP databases (https://db.cngb.org/blast4onekp/) using *Arabidopsis* gene models. Gene trees were rooted using *Chlamydomonas reinhardtii* and *Welwitschia mirabilis*. For D1/D2, *Gloeobacter kilaueensis* oxygen-evolving proteins were used as an outgroup. Multiple protein alignments and phylogenetic trees were generated using EBI-Clustal Omega (http://www.ebi.ac.uk/Tools/msa/clustalo/). Photoreceptor proteins phytochrome (PHY), cryptochrome (CRY), and phototropin were extracted from the 1KP database and compared with NCBI’s RefSeq database. The investigation focused on shade-tolerant and shade-intolerant characters of the conifers.

Conifer phenylalanine ammonia lyase (PAL) genes were extracted from ConiferDBMagic (Lorenz *et al.* 2012) and orthologs for the PAL genes were identified in the sequenced Douglas-fir genome. Protein alignments were executed with MAFFT (L-INS-i algorithm) and poorly aligned regions were block eliminated. For phylogenetic analysis of the PAL genes, sequences were from *Arabidopsis thaliana*, *Cucumis sativus*, *Glycine max*, and *Populus trichocarpa* (forming the dicot clade), *Brachypodium distachyon*, *Oryza sativa*, *Setaria italica*, *Sorghum bicolor*, and *Zea mays* (the monocot clade), *Pinus taeda*, *P. palustris*, *P. sylvestris*, *P. lambertiana*, *Picea abies*, and *Pseudotsuga menziesii* (gymnosperms), and *Physcomitrella patens* (bryophyte outgroup). For phylogenetic analysis, codon alignments were executed with PRANK (Löytynoja 2014). Following alignments, model estimation was performed with jModelTest (Darriba *et al.* 2012) and based on the AIC and tree consensus approach. Reconstruction of the PAL family phylogeny was completed using the IQTree available from http://www.cibiv.at/software/iqtree/ (Nguyen *et al.* 2015) with 1000 bootstrap replicates. Phylogenetic trees have been visualized using the iTOL interactive tree of life tool of the European Molecular Biology Labs (http://itol.embl.de/).

### Data availability

The *Pseudo*tsuga* menziesii* genome assembly has been deposited at NCBI as accession LPNX000000000 in BioProject PRJNA174450. Raw sequence data have been deposited in the NCBI SRA database under accession SAMN03333061. File S1 consists of supplemental material with sections on *Methods* and *Results* with additional tables (Tables S1–S4, S6, S7, and S13–S17 in File S1). File S2 consists of the supplemental figures (Figures S1–S15 in File S2). File S3 consists of a spreadsheet file with the remaining supplemental tables as tabs (Tables S5 and S8–S12 in File S3).

## Results and Discussion

### Whole-genome sequencing and validation of paired-end libraries

All paired-end and mate-pair sequencing was performed on a HiSeq2500 in Rapid Run mode producing reads that were uniformly 151 bp in length (Tables S1 and S2 in File S1). The Illumina Nextera Long Mate-Pair libraries contained a recognizable junction tag. For additional specificity, all mate-pair sequences were processed through NextClip (Leggett *et al.* 2014). Only reads containing a recognized junction fragment (NextClip categories A, B, and C) were presented to downstream mate-pair processing. This procedure filtered 482 million reads from the assembly, reducing the total number of mate-pair reads from 1182 million to 700 million. NextClip also reported a PCR duplication rate based on its estimation of read pairs previously observed in the library. While this information was not used for assembly, it was used to monitor the sequenced library complexity keeping it near or below a PCR duplication target of 10–15%. Total distinct clone coverage in mate-pair libraries presented to the downstream filtering and assembly was estimated by NextClip to exceed 250×, even after MaSuRCA filtering (Table S2 in File S1). This represents by far the deepest “clone coverage” achieved for any conifer assembly. To assess the paired-end data *k*-mer histograms were constructed using jellyfish (Marçais and Kingsford 2011). The 24-mer histogram for the target megagametophyte is presented in Figure S1 in File S2, with a dominant peak at a depth consistent with a haploid genome sequenced to our target depth. Additional smaller peaks at twice and three times the expected depth are also observed, consistent with a conifer genome comprised of ancient (diverged) copies of transposable elements. Using the method detailed by Zimin *et al.* (2014), four estimates of the genome size were computed using the 24-mer and 31-mer depth distributions and two methods of estimating the expected *k*-mer depth. All four genome size estimates were in close agreement, falling between 16.1 and 16.2 Gbp.

### Genome assembly

**Figure 1:**
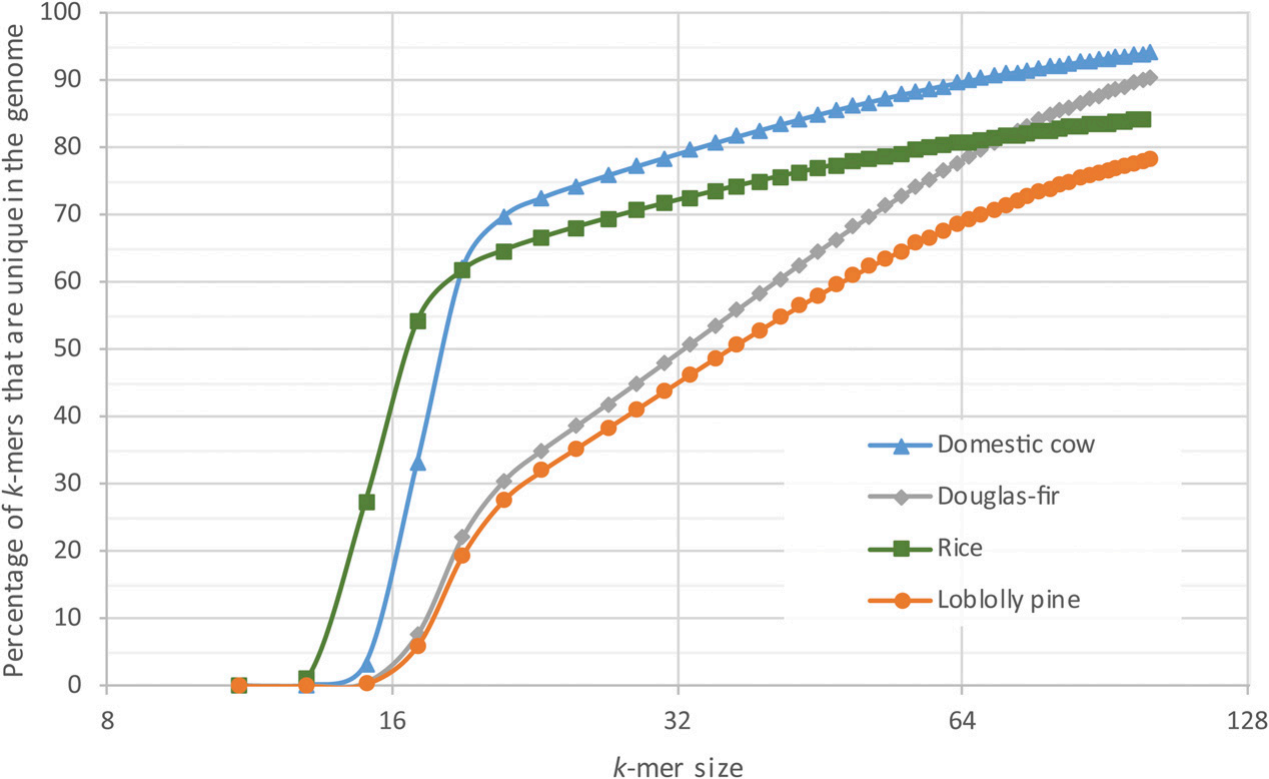
Comparison of the *k*-mer uniqueness ratio in the assemblies of *Pseudotsuga menziesii* (Douglas-fir), *Pinus taeda* (loblolly pine), *O. sativa* (rice), and *B. taurus* (domestic cow). Shown is the percentage of sequences of length *k* (*k*-mers) in each genome that occur exactly once (*i.e.*, that are unique) as a function of the value of *k*. Curves that are lower in the figure are relatively more repetitive and thus more difficult to assemble.

The final assembly, designated Pm_1.0, is characterized in Table 1. The N50 scaffold size is 340,704 bp, based on an estimated genome size of 16 Gbp; *i.e.*, 8 Gbp are contained in scaffolds whose size is 340,704 or larger. Including singletons (contigs not scaffolded), the total sequence content (scaffolds plus singletons) of the assembly is 15.7 Gbp. The Pm_1.0 assembly contains substantially larger contigs than those of the loblolly pine assembly (Neale *et al.* 2014; Zimin *et al.* 2014). The N50 contig size for Douglas-fir is 44 kbp, compared to an N50 size of just over 8 kbp for loblolly pine. The potential contribution of inherent differences in the repetitive nature of the genomes to the observed difference in contiguity was investigated. The relative repetitiveness of the two conifers as well as two unrelated genomes, *O. sativa* (rice) and *Bos taurus* (domestic cow), were analyzed. Their repetitiveness was measured with the *k*-mer uniqueness ratio (Schatz *et al.* 2010), a metric that uses the percentage of a genome that is covered by unique (single-copy) *k*-mers. As Figure 1 shows, for the relatively nonrepetitive cow genome, 80% of the genome is covered by unique *k*-mers of length 32. The rice genome is more repetitive, with just over 70% of the genome covered by unique *k*-mers of length 32. In contrast, at *k* = 32, only 50% of the Douglas-fir genome and 45% of the loblolly pine genome are covered. Note that because the analysis is performed on draft assemblies, repeats are likely to be underrepresented, thus the percentage of unique *k*-mers shown is an overestimate. As *k* gets large, it was observed that the percentage of unique *k*-mers in the Douglas-fir assembly approaches the percentage observed for mammalian genomes and diverges further from that of loblolly pine, providing more evidence that the Douglas-fir genome has fewer exact repeats. For all *k* > 20 in Figure 1, the Douglas-fir assembly is greater in size than the loblolly pine assembly. To generate the contigs for the assembly of Douglas-fir, *k*-mers of length 79 were used and the greater *k*-uniqueness ratio likely contributes to the much larger contig size in this assembly as compared to that of loblolly pine. Note also that this result based on the assembled conifer genomes is corroborated by examining the unassembled paired-end data. The relative area under the single mode peak in Figure S1 in File S2 represents the fraction of *k*-mers that are unique in the genome (*k*-mer uniqueness ratio). In Douglas-fir, this represents 43% for 31-mers, while for the much larger sugar pine genome (Figure S1 in File S2 in Stevens *et al.* 2016) this represents 40% for 31-mers.

**Table 1:** Summary statistics for Douglas-fir assembly version Pm_1.0

Feature	Contigs	Scaffolds	Singletons
Number of elements	3,541,350	2,814,118	6,349,354
Maximum size (bp)	692,189	3,893,220	150
Total size (bp)	14,647,181,470	14,947,693,430	746,567,730
N50 size (bp)	44,136	340,704	

All contigs and scaffolds longer than 150 bp were included in the statistics shown here. N50 sizes are computed based on an estimated total genome size of 16 Gbp. Singletons are small contigs that did not overlap with the rest of the assembly.

### Tandem and interspersed repeat analysis

Tandem repeat content was estimated at 1.7% after filtering for interspersed repeats. A total of 3785 unique interspersed elements were identified across 71.7% of the genome (scaffolds > 400 bp). Full results of repeat analysis are provided in the *Results* section in File S1.

### Gene space and completion assessment

A total of 54,830 gene models were reported by MAKER-P after multiple runs to improve gene prediction and subsequent improvements implemented by EvidenceModeler (section A of Table 2). Of these, 17,411 multi-exonic genes were supported by the assembled RNA-Seq data reflecting primarily needle tissue (Cronn *et al.* 2017). Additionally, several gene features were used to categorize the models, including: assignment of multi-exonic or mono-exonic status, support by at least one protein domain (or full-length protein evidence), and identification of canonical start and stop codons (full length). These filters generated a total of 34,239 high-quality multi-exonic genes of which 20,616 are full length (section B of Table 2). For mono-exonic genes, as a result of the prevalent pseudogene contents, greater confidence is found in a subset of 1641 which have a valid protein domain, RNA-Seq support, protein evidence, and full length. The combination of both yielded 22,257 high-quality, full-length gene models. Inclusion of high-quality partials brings this total to 35,880. Statistics on all gene-model sets (Table S3 in File S1) were calculated, as well as statistics of intron/exon splice junctions (Table S4 in File S1). Results reflect features that Douglas-fir has in common with other conifers and land plants in general: intronic space accounts for the majority of the gene space, when compared to exonic, having similar individual exon lengths with an average of over 200 bp and a maximum of 8000 bp, and the majority of splice junctions correspond to the canonical GT/AG. Average intron size (2301 bp) is longer than that for angiosperms (*e.g.*, *A. thaliana* 182 bp, *Populus trichocarpa* 366 bp, and *Vitis vinifera* 933 bp), but shorter than that for both *Pinus lambertiana* (8039 bp) and *P. taeda* (12,875 bp) (Stevens *et al.* 2016).

**Table 2:** Summary of Douglas-fir gene models (assembly Pm_1.0)

Category	Gene-model count
A – Single gene-model features used for classification	
Multi-exonic	47,874
Full-length	28,403
Expression	17,411
InterPro	36,243
Protein evidence	41,609
Mono-exonic	6956
Full-length	6874
Expression	1649
Interpro	6956
Protein evidence	6944
B – Gene-model classification based on combined features	
Multi-exonic	
Total high quality (expression/Interpro/ protein evidence)	34,239
Full-length	20,616
Partial	13,623
Mono-exonic	
Total high quality (expression/Interpro/ protein evidence)	
Full-length	1641
Total high quality full-length (Multi- and mono-exonic)	22,257
Total high quality (Multi- and mono-exonic)	35,880
Total low quality (Multi- and mono-exonic)	18,950

InterPro. gene model containing at least a recognizable protein domain; expression, gene model supported by RNA-Seq data corresponding to *de novo* assembled Douglas-fir transcripts; protein evidence, gene model with protein sequence similar to existing plant proteins in public databases (used as input for MAKER-P).

Informative functional annotations were found for 83% of the gene models with 31,939 of those assigned to at least one Molecular Function Gene Ontology term. Two independent approaches to assess gene-space completeness were evaluated: genome targeted via CEGMA which resulted in 95.2% completeness of the ultraconserved 248 CEGs and proteome targeted completeness via DOGMA and BUSCO (Table 3). BUSCO analysis revealed a total of 299 conserved single-copy orthologs. As compared to the CEGMA results, BUSCO revealed a lower conservation of the single-copy orthologs. BUSCO uses a set of the evolutionary informed single-copy orthologs from OrthoDB v9 whereas DOGMA uses a set of the PFAM-modeled evolutionary-conserved set of the conserved protein domains. Careful examination revealed the same pattern across the most recent annotation of *Pinus taeda*, whereas *Pinus lambertiana* as well as *Picea abies* revealed higher conservation in BUSCO. As part of the further assessment, the completeness of the protein domains via DOGMA was examined, which revealed greater conservation of the protein domains.

**Table 3:** Assessment of proteome completeness (%) using DOGMA and BUSCO approaches

Approach/domain	DF HQ	DF All	PT HQC	PT HQ All	PL HQC	PL HQ All	PA MC	PA HC
DOGMA								
CDA Found1	464	774	149	344	525	741	580	550
CDA Found2	272	471	97	229	280	431	141	287
CDA Found3	172	291	40	115	123	242	29	188
Total Found CDA	908	1536	286	688	928	1414	750	1025
Total % completeness	45	76	14	34	46	70	37	51
BUSCO								
Complete	299	523	216	321	466	593	107	455
Single	184	355	161	236	383	468	76	318
Multi	115	168	55	85	83	125	31	137
Fragment	203	283	144	193	148	188	242	230
Missing	938	634	1080	926	826	659	1091	755

DOGMA is the 965 single-domain CDAs and 1052 multiple-domain CDAs (Conserved Domain Arrangements) across eukaryotes and BUSCO is the Benchmarking Universal Single-Copy Orthologs. Explanation of headings: DF, Douglas-fir; PT, *Pinus taeda*; PL, *Pinus lambertiana*; PA, *Picea abies*; HQ, High Quality; HQC, High-Quality Complete; MC, Medium Content; HC, High Content.

### PAL genes

**Figure 2:**
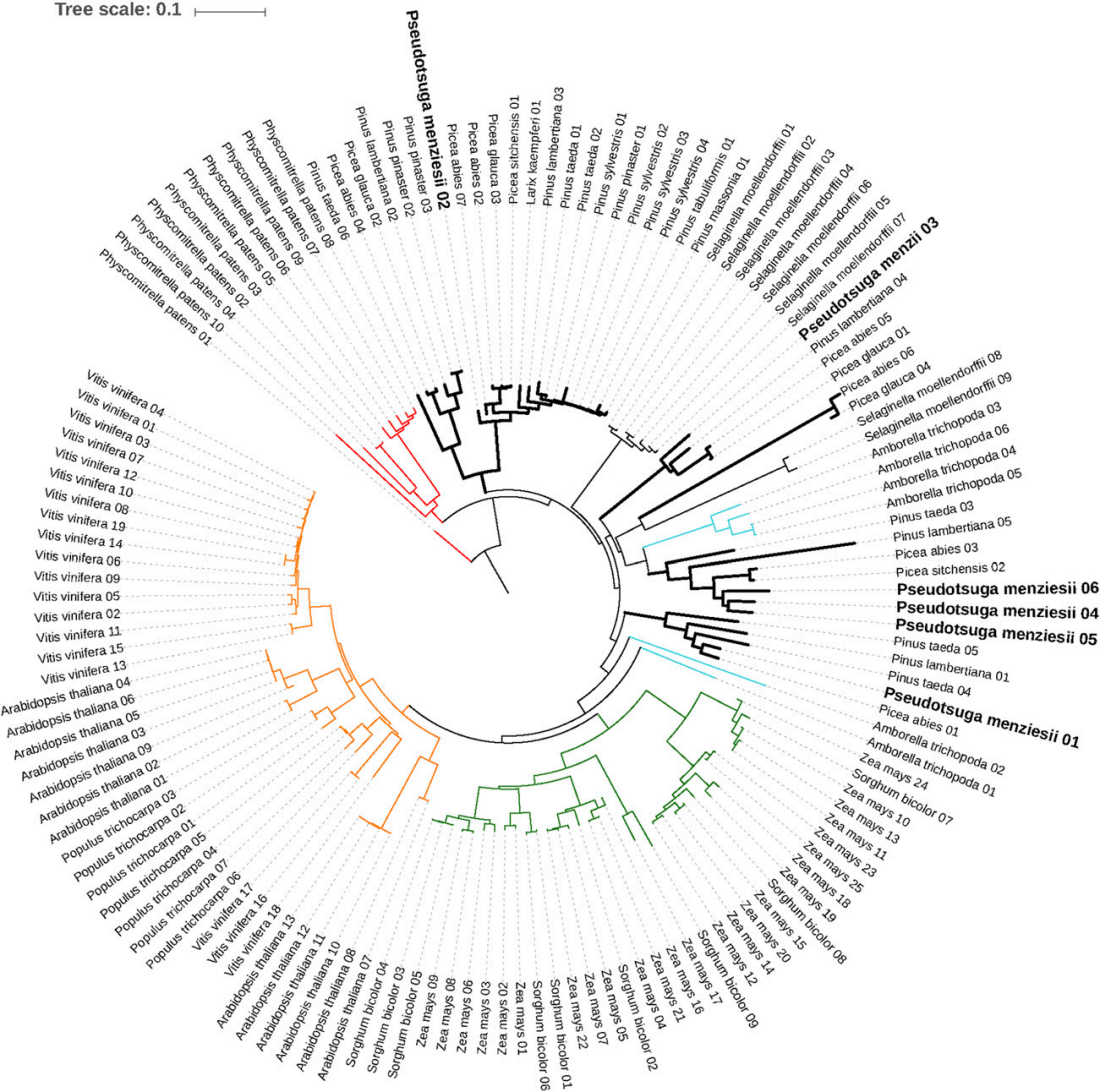
Phylogenetic placement of the PAL genes across land plants (dicots, monocots, lycophytes, and gymnosperms, with bryophytes as an outgroup). The green clade represents monocots (angiosperms), orange represents dicots (angiosperms), and the thick black line represents gymnosperms (signature of lineage-specific duplication). The conifer clade is bisected by the lycophyte *Selaginella moellendorfii* and the basal angiosperm *Amborella trichopoda* (cyan) indicative of early divergence and subsequent convergence in gene functions. The bryophyte *Physcomitrella patens* (outgroup) is shown in red. An interactive version of the tree is available at http://itol.embl.de/tree/1379989172117161489685231#.

Based on the abundance of myeloblastosis (MYB)-related transcription factor families (see the *Results* section in File S1), a phylogenetic placement was conducted using the rate-initiation enzyme PAL, which catalyzes the first enzymatic reaction in the phenylpropanoid pathway. Multiple sequence alignments of the PAL genes from the Douglas-fir genome and related species (Figure S2 in File S2) revealed conservation across motifs, including the MIO region (Al-Ser-Gly triad), suggesting convergence of the PAL genes in conifers. However, previous reports in *Pinus taeda* suggest a gymnosperm-specific lineage of PAL genes (Bagal *et al.* 2012). Based on the AIC criteria and bootstrap values, the gene-duplication events in the Douglas-fir genome appear to be lineage specific, which is in accord with previous reports and supports the gymnosperm-specific lineage of PAL genes (Bagal *et al.* 2012) (Figure 2). Annotation of the secretome, transcription factors, and phylogeny of the phenylpropanoid pathway, especially the rate-limiting enzyme (PAL gene), will enable further characterization of the metabolic system and play a role in developing strategies for characterizing the defense mechanisms of Douglas-fir.

### Gene turnover rates unrelated to whole-genome duplications are higher in Pinaceae than in dicots and monocots

**Figure 3:**
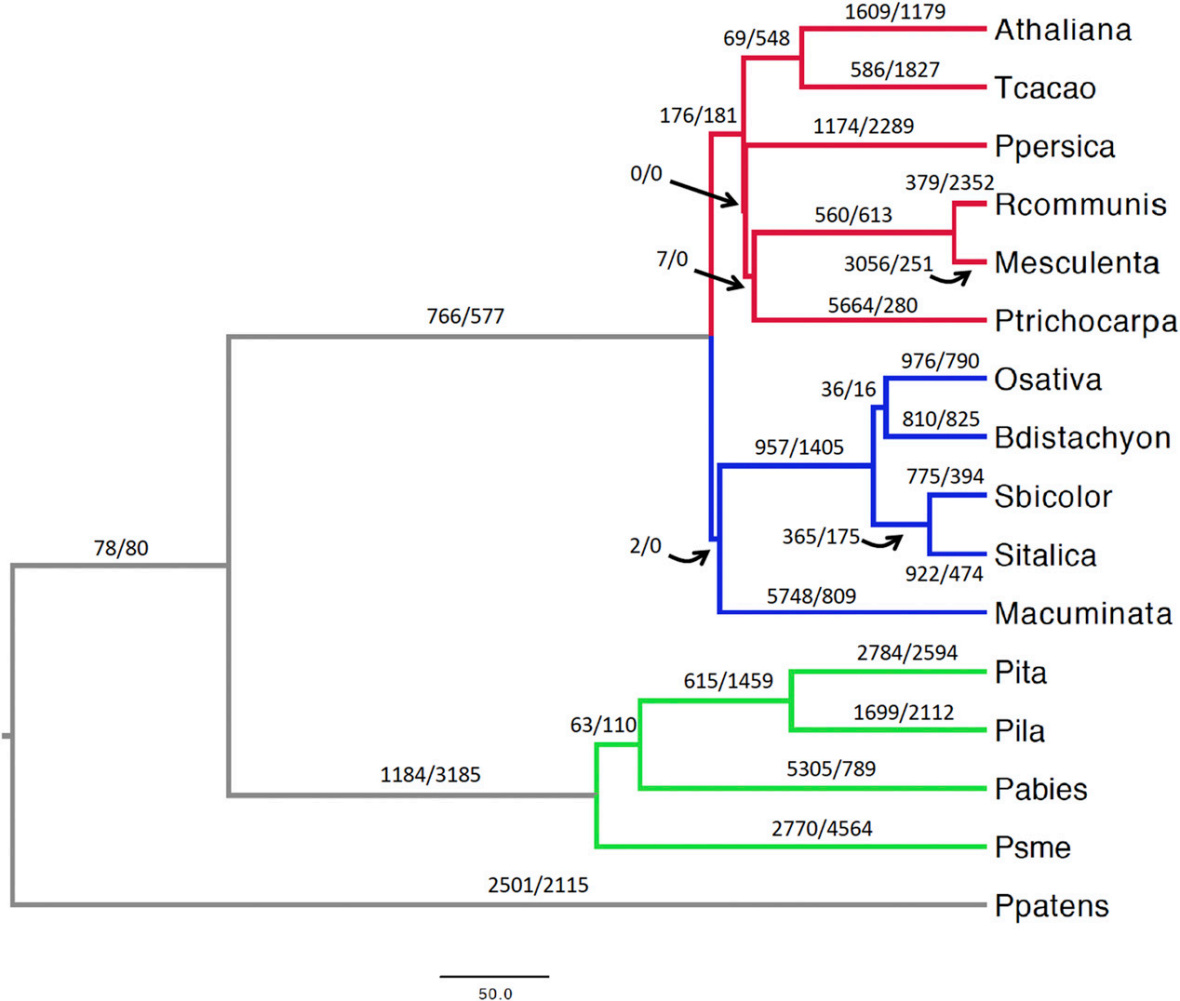
Gene-family evolution in 16 land plants. The red, blue, and green branches correspond to dicots, monocots, and Pinaceae, respectively. The paired numbers separated by a slash positioned on or nearby each branch indicate gene duplications (left of slash) and gene losses (right of slash). The scale bar is in million years. Athaliana, *A. thaliana*; Tcacao, *Theobroma cacao*; Ppersica, *Prunus persica*; Rcommunis, *Ricinus communis*; Mesculenta, *Manihot esculenta*; Ptrichocarpa, *Populus trichocarpa*; Osativa, *O. sativa*; Bdistachyon, *B. distachyon*; Sbicolor, *Sorghum bicolor*; Sitalica, *Setaria italica*; Macuminata, *M. acuminata*; Pita, *Pinus taeda*; Pila, *Pinus lambertiana*; Pabies, *Picea abies*; Psme, *Pseudotsuga menziesii*; Ppatens, *Physcomitrella patens*.

The rates of gene-family evolution (gene turnover) were investigated through the CAFE package (Han *et al.* 2013). A total of 6459 gene families shared across 16 land plants, corresponding to 230,055 genes (Table S5 in File S3), were examined. Two models were compared allowing for either a single λ or four λ values across the land plants tree (Figure 3). In the four-λ model, independent rates of gene turnover were estimated for dicots, monocots, Pinaceae, and their ancestral branches, and *Physcomitrella patens* (Ppatens, Figure 3). This model performed better than the single-λ model in all the CAFE runs and was applied in subsequent analyses. Gene turnover rates were 1.35 to 1.64 times higher for Pinaceae than for dicots and monocots (Table S6 in File S1). To account for the variation in gene annotation accuracy across species, the global error rates across the phylogeny were determined. This error accounts only for changes of one gene, either as a loss or gain (Han *et al.* 2013). Nevertheless, this model outperformed the nonerror model and provided an estimated global error rate of 0.07, implying that at least 7% of gene families contained an incorrect number of genes. Including this error rate in the analysis led to slightly decreased λ values for dicots, monocots, and Pinaceae, leaving the overall differences in gene turnover across these groups unaltered (Table S6 in File S1). A further model was implemented to infer species-specific error rates. Estimated error rates varied from zero to 0.3796875, with average error rates in Pinaceae ∼1 order of magnitude higher than in dicots and monocots (Table S7 in File S1). Remarkably, λ values were nearly identical in this model compared to the global error model, supporting the robustness to gene annotation errors of the observed accelerated gene turnover rate in Pinaceae compared to angiosperms (Table S6 in File S1). It is important to note that these gene turnover rate estimates do not take into account the massive gene duplications and subsequent gene losses associated with whole-genome duplications (WGDs). Because all examined angiosperm lineages experienced at least one polyploidization event in the past 100 MY (Li *et al.* 2016), while conifers may have undergone a single WGD before 210 MYA (Li *et al.* 2015), the overall frequency of gene duplications and gene losses is likely to be considerably higher in angiosperms than in Pinaceae.

Ancestral node family sizes were obtained from the four-λs CAFE run that included the estimated global error rate and these were used to infer the number of gene duplications and gene losses on each branch of the phylogeny (Figure S3 in File S2). Some gene-family expansions clearly reflect the larger gene sets used in certain species, for instance, in the branches leading to black cottonwood (*Populus trichocarpa*) and banana (*Musa acuminata*). With the exception of *Picea abies*, Pinaceae exhibited the lowest number of annotated genes in these families; accordingly, the highest number of gene-family contractions was found for loblolly pine, Douglas-fir, and the Pinaceae branch (Figure S3 in File S2 and Table S5 in File S3). This annotation bias implies that gene duplications in the Pinaceae branch are underestimated. Therefore, the 529 gene families that expanded along this branch are more likely to represent the result of selection toward retention of gene duplicates rather than an artifact due to mis-annotated genes (Figure S3 in File S2). Douglas-fir showed the highest number of contracted families and overall gene losses across the tree, possibly due to genome fragmentation and related partial annotations (Table S5 in File S3).

### Rapidly evolving gene families and genetic networks in Douglas-fir

The Viterbi algorithm integrated in CAFE estimated 671 rapidly evolving gene families at a p-value ≤0.05 across the land-plant phylogeny (Figure 3 and Tables S5 and S8 in File S3). In Pinaceae, 351 families showed significant variation in at least one branch. Despite the tendency toward gene loss in Douglas-fir, 80% of the 108 gene families with a significant gene turnover rate in Douglas-fir represented lineage-specific expansions due to gene duplications (Figure S3 in File S2 and Tables S5 and S8 in File S3). A genetic network analysis revealed several networks associated with either gene losses or duplications in the 108 gene families with high turnover rates in Douglas-fir. To minimize possible biases due to the incomplete gene annotation in Douglas-fir, two additional filtering steps on the genetic network results were performed. First, Douglas-fir gene families with average coding-sequence length <90% of the average coding-sequence length in *A. thaliana* genes in the same family were removed. This step minimizes the contribution of gene fragments (pseudogenes) commonly found in Pinaceae genomes to gene-family size increase. Second, only the genetic networks where at least two gene families showed signatures of contraction or expansion in Douglas-fir were included, following the reasoning that simultaneous gene losses or duplications across different families involved in the same network are the result of underlying natural selection acting upon those pathways rather than the result of spurious gene annotation. As a result of applying these criteria, three genetic networks with significant gene losses and three networks with significant duplications in Douglas-fir were identified (Tables S9 and S10 in File S3).

### Comparison of all gene families shows gains and losses of genetic networks in Douglas-fir and Pinaceae

To gain further insights into the possible biological impact of gene duplications and losses in conifers, changes between angiosperms and Pinaceae across all gene families were identified, including those that were not used in the CAFE analyses. Comparison of the size of each gene family between angiosperms and Pinaceae confirmed the overall trend of higher rates of family contraction in the latter, as expected given the lower number of annotated genes in pine trees and Douglas-fir (Table S11 in File S3). A total of 876 gene families were entirely lost in Pinaceae as opposed to 281 families absent in angiosperms, a difference that is at least in part due to the higher number of flowering plant species used in these analyses (see *Materials and Methods*). Pinaceae also exhibited 10 times more gene families with a contraction compared to angiosperms, and fewer lineage-specific family expansions than flowering plants (Table S11 in File S3). Gene ontology analyses showed a variety of processes and functions with significant enrichment across lineages.

These analyses revealed expanded gene families in Douglas-fir and gene families that have been lost in Pinaceae. Conifers are capable of regulating defense through the phenolic and terpenoid biosynthesis process. Constitutive regulation of these pathways allows for the phloem defense responses. Previously, it has been widely demonstrated that jasmonate-induced ethylene production favors the conifer defense system (Hudgins and Franceschi 2004). As with the CAFE analyses, genetic networks wherein multiple gene families experienced independent losses or duplications were identified. Two large genetic networks were expanded in Douglas-fir, including the jasmonate-regulation network identified through the CAFE results (Table S12 in File S3). Observance of retention of the key regulators of the jasmonate-signaling pathways supports the previous observation. Both expanded networks in Douglas-fir contained genes from seven families, suggesting selection for retention of gene duplicates. Several apparent gene losses in Pinaceae involved photosynthesis-related processes. More than 100 such genes are connected in a large genetic network in *A. thaliana* (Table S12 in File S3). Analyses of four Pinaceae genomes and transcriptomes confirmed that at least 27 of these genes have been lost in Pinaceae after they separated from Cupressaceae (Table S12 in File S3). The majority of these genes represent either subunits or cofactors of the chloroplast NDH-dehydrogenase complex involved in the cyclic electron transport at the PSI level. We found that LHCa5, a component of the PSI, is also absent in Pinaceae.

### Evolution of the NDH-complex in Pinaceae: insights from Douglas-fir comparative genomics

Previous studies have shown that multiple NDH-complex proteins encoded in the chloroplast are absent in the genome of this organelle in Pinaceae (Wakasugi *et al.* 1994; Long *et al.* 2008; Braukmann *et al.* 2009). Whereas chloroplast genome sequences from conifers suggested that Pinaceae lost all chloroplast NDH-complex genes, it remained unclear whether nuclear genes encoding proteins of the NDH-complex are also absent in these conifers. In our study, we demonstrate that 18 nuclear NDH-complex genes have also been lost in Pinaceae, whereas only three NDH-complex genes are likely absent in both Pinaceae and Cupressaceae (Table S12 in File S3). Both the NDH-complex and the PGR5/PGRL1 pathway are responsible for the cyclic electron transport around the PSI system (Shikanai 2014). We confirmed that both PGR5 and PGRL1 orthologous genes occur in Pinaceae (data not shown); therefore, the cyclic electron transport process is likely still active in Pinaceae, albeit by means of the single PGR5/PGRL1 pathway. Angiosperm mutants lacking NDH-complex functionality show significant impairment of photosynthesis only in oxidative stressful conditions for the chloroplast (Endo *et al.* 1999; Wang *et al.* 2006). Pinaceae leaves might have evolved specific anatomical, physiological, and molecular adaptations that led to a relaxation of the selective pressure on the NDH-complex genes and their loss. Intriguingly, the loss of plastid NDH-complex genes has also been reported in the gymnosperm lineage Gnetales (Braukmann *et al.* 2009), as well as in both parasitic and carnivorous angiosperms (Bungard 2004; Wicke *et al.* 2014). Chloroplast NDH-complex genes are lost in parasitic angiosperms before the pseudogenization of genes involved in photosynthesis light reactions, indicating that overall the NDH-complex might be dispensable under certain evolutionary scenarios. Whereas the emergence of complete or partial heterotrophy throughout parasitism and predation is likely the driving force behind the loss of NDH-complex genes in angiosperms, the possible advantages of NDH-complex-free chloroplasts in Pinaceae and Gnetales are less evident. Finding the evolutionary drivers of the NDH-complex dispensability in these two gymnosperm groups may provide new insights into the long-standing debate regarding the phylogenetic position of Gnetales within gymnosperms.

### Shade tolerance, photoreception, and light-harvesting complexes in conifers

**Figure 4:**
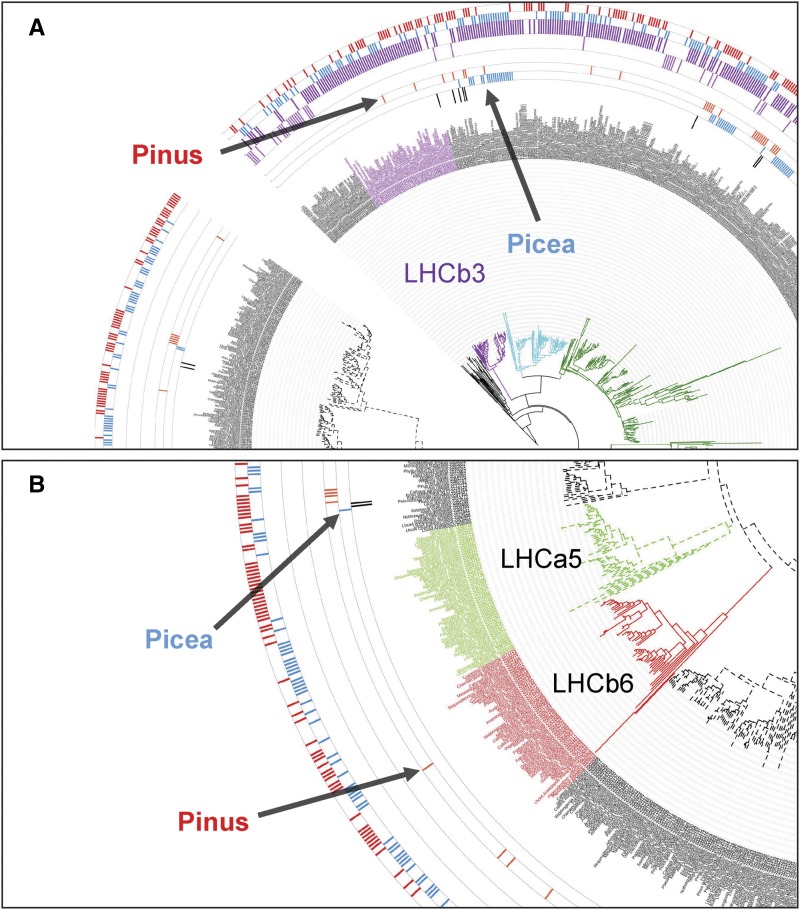
Enlarged excerpts from a phylogenetic tree comparing Douglas-fir and other conifers together with select angiosperm light-harvesting-complex proteins. The full tree is presented in Figure S4 in File S2 and an interactive version is available at http://itol.embl.de/tree/1379989172344871490022208#. Members of the genera *Pinus* (pink) and *Picea* (blue) are shown in the two innermost data bars in both excerpts, circling the labels. Featured here are antenna PSII protein LHCb3 (purple clade) in (A) and PSII LHCb6 (red clade) and PSI LHCa5 (olive green clade) in (B). (A) None of the members of the genus *Pinus* nor Douglas-fir have proteins that cluster with the LHCb3 clade. (B) The same trend is observed for the LHCb6 and PSI LHCa5 proteins. These proteins may have been lost in most conifers, but this is not a pan-conifer event.

Ancestral aquatic cyanobacteria evolved two photosystems (PSI and PSII) as initial steps of photosynthesis. Both photosystems have structurally conserved core complexes bearing a reaction center surrounded by a variable peripheral antenna system with light-harvesting complexes (Caffarri *et al.* 2014). Douglas-fir is a well-noted example for shade intolerance and its light-harvesting systems may have evolved to perform solely in high-light environments. Such an adaptation could eliminate the risk of photo-damage while transitioning from low- to high-light conditions. The constitutively high-light-adapted nature of light-harvesting complexes in Douglas-fir is evident from the inability of seedlings to establish under closed-canopy conditions. Accordingly, the excitatory diffusion length and quenching center organization may be permanently locked in the dissipative state (Amarnath *et al.* 2016). An early expression study has provided support for the constitutive expression of light-harvesting antenna protein genes in dark- and light-adapted Douglas-fir seedlings (Alosi *et al.* 1990). Concomitant absence of LHCa5 in PSI and the two PSII antenna proteins LHCb3 and LHCb6 could be an adaptive means to maintain loose non-array-forming light-harvesting complexes in dissipative mode. Our analysis containing 77 conifers, three Gnetales, and nine angiosperm light-harvesting complex proteins generated a tree with 1102 leaves (Figure S4 in File S2) where all six PSII and five PSI proteins are represented. In this tree, no pine or Douglas-fir proteins are identified within the LHCb3 (Figure 4A) and LHCb6 (Figure 4B) clades suggesting that the two proteins have been lost in those genera. Photoprotection by nonphotochemical quenching (NPQ) and state transitions between PSI and PSII have been reported to be affected by LHCb6, LHCb5, and LHCb4. Knockout mutants of the three proteins in *Arabidopsis* have shown that the two quenching centers (Q1/Q2) observed in wild-type plants undergo a reorganization. Absence of LHCb6 leads to dissociation of Q1 even in dark conditions and Q2 becomes a pre-high-light-adapted PSII quenching center (Miloslavina *et al.* 2011). Thus, absence of LHCb6 could be a contributing factor toward a shade-intolerant phenotype. In *Chlamydomonas* and *Rhodospirillum photometricum*, high-light-adapted photosynthetic membranes show wide spacing among PSI and PSII mediated by mobile light-harvesting complex proteins (Scholes *et al.* 2011). Time-resolved fluorescence microscopy of wild-type *Chlamydomonas* has revealed that PSII, LHCII, and PSI remain detached under high-light conditions (Ünlü *et al.* 2014). Similarly, with the lack of many members of light-harvesting proteins from both photosystems, Douglas-fir may be emulating the land-plant version of the dissipative light reactions.

One of the perils of being a terrestrial plant exposed to high light is the risk of damage to the homologous reaction center cofactor proteins D1 and D2 where charge separation and water oxidation are coordinated. The D1 protein provides most of the ligands to the water-splitting Mn_4_CaO_5_ residue (Cardona *et al.* 2015). Douglas-fir possesses at least three D1 paralogs based on transcriptomic evidence (Figure S5 in File S2) which may be an adaptation as an enhanced repair mechanism increasing the turnover of this most vulnerable reaction center protein.

Shade tolerance in ferns has been shown to be conferred through a chimeric neochrome photoreceptor formed by fusion of red/far-red-sensing phytochrome and blue-light-sensing cryptochrome, which provides a competitive advantage to this early land-plant lineage in angiosperm-dominated closed canopies (Li *et al.* 2014). Our investigation of the three photoreceptor proteins phytochrome (PHY), cryptochrome (CRY), and phototropins (PHOT) did not indicate a specialized adaptation among shade-tolerant and shade-intolerant gymnosperms in terms of photoreception (Figures S6–S8 in File S2).

The analysis was extended to include proteins and enzymes that are mediators of state transitions including pH sensor protein PsbS, violaxanthin de-epoxidase VDE1, the H subunit of PSI (PSAH1), and thylakoid-associated kinases STN7 and STN8 (Figures S9–S12 in File S2) (Kargul and Barber 2008). When species are labeled based on their shade tolerance/intolerance phenotype, no significant clustering was observed with both phenotypes represented on clades in an intercalated manner (Figures S9–S12 in File S2). The pattern observed in gene trees constructed for the photoreceptor proteins PHY, CRY, and PHOT was the same. Based on CAFE analysis, STN8, which phosphorylates PSII core proteins, was absent in Douglas-fir, which could be a contributing factor for shade intolerance. The intercalated representation of the proteins belonging to both shade-tolerant and shade-intolerant species suggests that shade tolerance in conifers may be conferred through a different mechanism tied to light-harvesting such as leaf morphology. It has been shown that southern hemisphere conifers, which are heavily represented in our gene trees, follow an angiosperm-like strategy involving flattened leaves and shoots. This kind of adaptation to angiosperm-dominated forests including in the tropics is especially noticeable among Podocarpaceae where maximal light-harvesting is combined with increased hydraulic conductance, both rather limited in needle-leaved conifers such as Douglas-fir (Biffin *et al.* 2012). Indeed, in our large compilation of light-harvesting proteins those that are reported to be lost in specific conifer lineages are represented among the southern hemisphere conifers (Figure S4 in File S2).

### Conclusions

The reference genome sequence for Douglas-fir reported here not only sheds additional light on the large anatomical, morphological, and physiological differences between angiosperms and gymnosperms but also begins to reveal the evolutionary changes that have occurred in the family Pinaceae. Species of Pinaceae can range from highly shade tolerant to highly shade intolerant. These differences are fundamentally important not only in natural forest succession processes, but also in artificial reforestation practices. Developing a deeper understanding of the genetic and physiological processes determining shade tolerance/intolerance will facilitate improved forest practices in plantations and natural forests.

## Supplementary Material

Supplemental material is available online at www.g3journal.org/lookup/suppl/doi:10.1534/g3.117.300078/-/DC1.
