# Supplementary material for: The Douglas-Fir Genome Sequence Reveals Specialization of the Photosynthetic Apparatus in Pinaceae
Source: G3 (Bethesda). 2017 Jul 27;7(9):3157–67. doi: 10.1534/g3.117.300078 (PMC5592940; doi:10.1534/g3.117.300078)
Supplement: Supplementary file 1 [file 3157S1.pdf]

The Douglas-fir genome sequence reveals specialization of the photosynthetic apparatus in Pinaceae

Supplemental File 1

METHODS

Repeat annotation

Scaffolds greater than 400 bp were used for repeat analysis. To detect simple repeats across the full genome, Tandem Repeat Finder (TRF) (Benson 1999) was executed with the following parameters: matching weight of 2, mismatch weight of 7, indel penalty of 7, match probability of 80, indel probability of 10, minimum score of 50, and a maximum period size of 2000. For accurate estimation and distribution of tandem repeats, those overlapping with interspersed repeats were filtered from the set. Tandem repeats were categorized by period size using the following thresholds: 2 to 8 bp as microsatellites, 9 to 100 bp as minisatellites, and >100 bp as satellites. Mononucleotides were removed due to the high likelihood of error in their determination. For elucidation of interspersed repeat elements, both similarity and *de novo* based approaches were applied. RepeatModeler combines two complementary *de novo* repeat element prediction algorithms, RECON (Bao and Eddy 2002) and RepeatScout (Price *et al.* 2005). For RepeatModeler, (3% of genome + fosmid) was used as the input set. For the whole genome set, a combination of TEclass (Abrusan *et al.* 2009), CENSOR (Kohany *et al.* 2006), and manual characterization was used to identify the uncharacterized elements from the *de novo* repeat library obtained from RepeatModeler. The *de novo* library, combined with the plant Repbase (Jurka *et al.* 2005) library (Virdiplantae, v19.01) was used as the reference database for RepeatMasker v4.0.5. Full-length elements were determined by applying a cut-off of 80-80-80 (80% sequence similarity and 80 bp minimum length) (Wicker *et al.* 2007).

MAKER genome annotation

Annotations for the assembled genome were generated using the partially automated pipeline MAKER-P (Campbell *et al.* 2014), which aligns and filters existing evidence, produces *ab initio* gene predictions, and infers 5' and 3' UTRs. The final gene set is a result of integrating these sources. Inputs for MAKER-P included scaffolds longer than 800 bp, protein homology evidence, expressed sequenced tags (ESTs), a custom repeat library, and models constructed with gene prediction algorithms. Protein evidence resulted from sequences at least 20 amino acids in length from six angiosperms: *Eucalyptus grandis*, *Oryza sativa*, *Vitis vinifera*, *Populus trichocarpa*, *Amborella trichopoda*, and *Physcomitrella patens*. An additional four gymnosperms were included: *Picea abies*, *Picea glauca*, *Pinus taeda*, and *Pinus lambertiana* (Table S13). The transcript evidence was generated through *de novo* assembly of *Pseudotsuga menziesii* (Cronn *et al.* 2017). Additionally, Genbank-sourced ESTs of *P. menziesii* were also included (Table S14). In order to gain a comprehensive set of possible genes, ESTs were collected from the following genera: *Picea*, *Pinus*, and *Cryptomeria* (Table S15).

Alternative EST sequences less than 150 bp in length were removed and those remaining were clustered with USEARCH (Edgar 2010) at 98% identity. There were 6,229 of the 479,372 alternative ESTS which were successfully aligned by GMAP to the *P. menziesii*

genome (90% coverage, 95% identity) (Wu and Watanabe 2005). A total of 36,991 of the 76,541 *P. menziesii* ESTs were successfully aligned to the genome (90% coverage, 98% identity). Protein alignment via Exonerate (Slater and Birney 2005) resulted in 56.5% of the gymnosperm and 2.0% of the angiosperm proteins being aligned to the genome (70% coverage). The resulting GFF3 alignments from GMAP and Exonerate were processed by MAKER-P.

Gene prediction tools, such as Augustus v3.0.3 (Stanke *et al.* 2008) and SNAP vsnap-2013-11-29 (Korf 2004) were implemented for the development of gene-identification parameters in the assembled genome. The alignments of protein and transcriptome evidence were used to train Augustus. A custom repeat library generated from *de novo* and similarity approaches was provided for masking. MAKER-P was run over three iterations with subsequent manual review to improve upon gene-model prediction.

Genome, proteome, and gene space completion analysis
The entire set of filtered gene models was evaluated for completeness. BUSCO was used with default parameters and the plant reference set (950 orthologs) (Simão *et al.* 2015). Completeness of the gene space relative to the genome was also analyzed with the Core Eukaryotic Genes Mapping Approach (CEGMA) (Parra *et al.* 2007) pipeline with default parameters. The ultra-conserved set of 248 Core Eukaryotic Genes (CEGs) was used as the reference. Finally, DOGMA (Dohmen *et al.* 2016) was applied, which predicts the proteome completeness based on the 965 single-domain CDAs (Conserved Domain Arrangements) and 1,052 multiple-domain CDAs across eukaryotes. In total, two sets of annotated gene models from each of the four conifer genomes were evaluated. Douglas-fir (22,257 high confidence (HC) and 54,830 total), *Pinus taeda* (4,690 HC and 8,775 total), *Pinus lambertiana* (8,775 HC and 13,936 total), and *Picea abies* (26,437 HQ and 32,150 MQ, corresponding to the HC and total gene categories, respectively, in the analyses for the other three species).

Functional gene annotation and features of assembled genome
Functional annotation of the filtered gene models was executed with the UBLAST tool of USEARCH to identify local alignments (v.7.0.1090, E-value threshold of 1E-9 and a weak E-value of 1E-3) (Edgar 2010). A custom set of curated plant proteins from the NCBI non-redundant database and the NCBI RefSeq Protein database were queried. Selection and assignment of the best annotation derived from the UBLAST alignments were performed with the Eukaryote Non-Model Transcriptome Annotation Pipeline (enTAP, https://github.com/SamGinzburg/WegrzynLab). Gene Ontology (Ashburner *et al.* 2000) terms were assigned for Molecular Function, Biological Process, and Cellular Component using Blast2GO v.3.2.7 (Conesa and Götz 2008). Douglas-fir high-quality (HQ) gene space was further analyzed for the identification of the transcription factors using the Plant TFcat available from http://plantgrn.noble.org/PlantTFcat/ (Dai *et al.* 2013) and compared with the plant transcription factor database (http://plntfdb.bio.uni-potsdam.de/v3.0/ and http://planttfdb.cbi.pku.edu.cn/). To predict and characterize secreted proteins, a comparative analysis was performed using several secretory prediction algorithms such as ChloroP (www.cbs.dtu.dk/services/ChloroP/), signalP (www.cbs.dtu.dk/services/SignalP/), TMHMM (www.cbs.dtu.dk/services/TMHMM/),

TargetP (www.cbs.dtu.dk/services/TargetP/), and Phobius (phobius.sbc.su.se/). In addition to these, selected proteins having a defined signal from the above algorithms were further scanned for ER (endoplasmic reticulum) signal and removed from downstream analysis if detected. Functional domains were assigned to the predicted core secretory proteins via HMMscan again the PFAM repository (E-value threshold of 1E-5).

Genome wide orthology and evolutionary analysis
For orthology searches, protein sequences from 17 species were downloaded from Phytozome version 10.0 (numbers of sequences are provided in parentheses): *Arabidopsis thaliana* (27,416), *Brachypodium distachyon* (31,694), *Glycine max* (56,044), *Manihot esculenta* (30,666), *Musa acuminata* (36,549), *Oryza sativa* (39,049), *Physcomitrella patens* (26,610), *Pseudotsuga menziesii* HQ (22,257), *Populus trichocarpa* (41,335), *Prunus persica* (27,864), *Ricinus communis* (31,221), *Sorghum bicolor* (33,032), *Setaria italica* (35,471), *Theobroma cacao* (29,452), *Picea abies* (19,607), *Pinus taeda* (21,346), *Picea glauca* (13,026), and *Pinus lambertiana* (33,113).

OrthoFinder v0.4.0 (Emms and Kelly 2015) was used to identify orthologous protein coding genes in the four Pinaceae species. The analyzed datasets included an expanded 84,988 putative proteins from *Pinus lambertiana*, 83,861 from *Pinus taeda*, 54,626 from *Pseudotsuga menziesii*, and 63,621 from *Picea abies* which were assessed for the Pinaceae-specific orthology assignments. OrthoGroups (gene families) in OrthoFinder are defined as homologous genes descended from a single gene from the last common ancestor of the species examined. It is assumed that a parental gene of each orthogroup was present in the common ancestor of the four Pinaceae species. This method accounts for gene length and phylogenetic distance between species. The algorithm is also robust to missing genes, a potential challenge in incomplete genome assemblies. Pre-computed NCBI blastp v2.2.29+ (Camacho *et al.* 2009) results were used as input for OrthoFinder. Additionally, the program mcl v14-137, an implementation of the Markov Cluster Algorithm (Van Dongen 2000; Enright *et al.* 2002) was used by OrthoFinder with the default inflation parameter of 1.5.

Gene-family evolution analysis with CAFE
To assess the evolutionary rate of gene families across seed plants and between Pinaceae, several analyses using CAFE v3.1 (Han *et al.* 2013) were performed. *Picea glauca* gene families were removed due to the few annotated genes for this species. Soybean families were additionally excluded due to an excess number of putative duplicate genes. Two datasets were analyzed with CAFE: a gene family dataset of land plants including six dicots, five monocots, four species of Pinaceae, and *Physcomitrella patens*, and a Pinaceae-only gene family dataset. To reduce possible biases due to gene mis-annotation and to ensure a broad phylogenetic representation, families of size zero in one or multiple major taxonomic groups: dicots, monocots, Pinaceae, and *P. patens*, were excluded. Furthermore, we removed gene families that differed by 100 genes or more between species with the lowest and highest gene count to prevent issues with the calculation of λ. Models with increasing complexity were applied, from one λ to multiple λ values across the phylogeny, to both datasets. For each model, at least five CAFE runs were performed and those runs with the highest likelihood value per model were included. Only models that showed convergence of

likelihood and λ values over multiple runs were used for downstream inferences on gene family evolutionary dynamics. Because of the variation across species in the completeness of their genome annotations, the models were refined by including estimates of error in gene family size. Global error models were calculated and incorporated in subsequent CAFE runs to obtain improved reconstruction of λ values and ancestral gene family sizes.

Analysis of all gene families
Median gene family sizes from angiosperms and Pinaceae were compared to obtain estimates of expansions, contractions, and losses of gene families in both lineages. Only gene families with at least one member in *P. patens* were investigated. Families with a larger median size in angiosperms than Pinaceae and *P. patens* were considered expanded in flowering plants; similar comparisons were made to infer expansions and contractions in Pinaceae. Gene families were considered lost in angiosperms or Pinaceae when no genes occur in the corresponding species while one or more genes occurred in the other seed plant lineage and in *P. patens*. Gene Ontology enrichment was calculated on the agriGO server (http://bioinfo.cau.edu.cn/agriGO/) using the Singular Enrichment Analysis (SEA) with default settings and the Plant GO slim database. Only one *A. thaliana* gene per family was used in this GO analysis. Gene networks were built using STRING (Szklarczyk *et al.* 2015) using all *Arabidopsis thaliana* genes in the analyzed gene families.

RESULTS

Tandem and interspersed repeat analysis
Of the tandem repeat content, as expected, minisatellites occupied the largest percentage of the genome (0.8%). This was followed by satellites which covered 0.7% of the Douglas-fir genome. Among minisatellites, 21, 20, and 24 bp covered the maximum portion of the genome. Of the interspersed repeat content, similarity-based hits (alignments to Plant Repbase) accounted for only 3.5% of the total, highlighting the scarcity of conifer elements characterized as well as the diverse nature of the content. A total of 15% of the repeats could be annotated as full-length while partial elements composed 56.7%. LTR retrotransposons constitute the majority of the repeat content as expected at 62.7% whereas DNA transposons constitute 7.0%. A detailed classification of the interspersed repeat is shown in Figures S13 and S14. Among LTRs, Gypsy elements constitute 24.8% of the genome whereas Copia elements constitute 11.8% resulting in a Gypsy to Copia ratio of 2.09:1. The top 20 LTR elements represent 20.3% of the genome (Table S16).

Secretomics and transcription factors
Among traits of biological relevance, carbon sequestration plays a vital role in tree sustainability by re-directing carbon to the production of phenolic compounds, which, in turn, are involved in disease resistance. Carbon sequestration occurs by reverse immobilizing carbon through the shikimate pathway and by regulating the transcriptional control of the phenylpropanoid pathway (Craven-Bartle *et al.* 2013; Liu *et al.*, 2015). The secretome describes many of the proteins involved in pathways responding to biotic and abiotic challenges. Accounting for the relationship of the secretome and transcriptional pathways, a total of 895 core secretory proteins were identified among the high-quality gene models (Table S17; Figure S15A). Functional assignment reveals genes containing

myeloblastosis (MYB) domains, DUF, FAD, and other lignin containing domains. Abundance of these domains highlights the role of the lignin metabolism and glycosyltransferases as important for cell wall maintenance and metabolism. MYB transcription factors, along with the bHLH family, play an important role in regulating the metabolic diversity (Feller *et al.* 2011). Genome-wide characterization of the transcription factors identified 2,698 high-quality gene models (Figure S15B), which is higher than the number found for *Picea abies* (1581), *P. glauca* (559), *P. sitchensis* (362), and *Pinus taeda* (442). The annotation of MYB-related transcription factors in Douglas-fir (296) compared to the 148 MYB families identified in *Picea abies* suggests an abundance of MYB-regulated pathways which are associated with lignin and phenylpropanoid pathways.

REFERENCES

Abrusan, G.; N. Grundmann, L. DeMester, W. Makalowski, 2009 TEclass—a tool for automated classification of unknown eukaryotic transposable elements. Bioinformatics 25(10): 1329-1330.

Ashburner, M., C. A. Ball, J. A. Blake, D. Botstein, H. Butler, *et al.*, 2000 Gene Ontology: tool for the unification of biology. Nature Genet 25: 25-29.

Benson, G., 1999 Tandem repeats finder: a program to analyze DNA sequences. Nucleic Acids Res. 27: 573–580.

Bao, Z.; S. R. Eddy, 2002 Automated de novo identification of repeat sequence families in sequenced genomes. Genome Res 12: 1269-1276.

Camacho, C., G. Coulouris, V. Avagyan, N. Ma, J. Papadopoulos, *et al.*, 2009 BLAST+: Architecture and applications. BMC Bioinformatics 10: 421. doi: 10.1186/1471-2105-10-421

Campbell, M. S., C. Holt, B. Moore, and M. Yandell, 2014 Genome annotation and curation using MAKER and MAKER-P. Curr Protoc Bioinformatics 48: 4.11.1-4.11.39. doi: 10.1002/0471250953.bi0411s48

Conesa, A. and S. Götz, 2008 Blast2GO: A comprehensive suite for functional analysis in plant genomics. International Journal of Plant Genomics 2008: 619832. doi: 10.1155/2008/619832

Craven-Bartle, B., M. B. Pascual, F. M. Cánovas, and C. Ávila, 2013 A Myb transcription factor regulates genes of the phenylalaline pathway in maritime pine. Plant J 74, 755–766.

Cronn, R., P. C. Dolan, S. Jogdeo, J. L. Wegrzyn, D. B. Neale, *et al*., 2017 Transcription through the eye of a needle: Daily and annual cycles of gene expression variation in Douglas-fir needles. bioRxiv 117374 https://doi.org/10.1101/117374

Dai, X., S. Sinharoy, M. Udvardi, and P. Xuechun Zhao, 2013 "PlantTFcat: An online plant transcription factor and transcriptional regulator categorization and analysis tool." BMC Bioinformatics 14(November): 321. doi: 10.1186/1471-2105-14-321

Dohmen, E., L. P. M. Kremer, E. Bornberg-Bauer, and C. Kemena, 2016 DOGMA: Domain-based transcriptome and proteome quality assessment. Bioinformatics btw231. doi: 10.1093/bioinformatics/btw231

Edgar, R. C., 2010 Search and clustering orders of magnitude faster than BLAST. Bioinformatics 26(19): 2460-2461.

Emms, D. M. and S. Kelly, 2015 OrthoFinder: Solving fundamental biases in whole genome comparisons dramatically improves orthogroup inference accuracy. Genome Biology 16: 157. doi: 10.1186/s13059-015-0721-2

Enright, A. J., S. Van Dongen, and C. A. Ouzounis, 2002 An efficient algorithm for large-scale detection of protein families. Nucleic Acids Res 30: 1575-1584.

Feller, A., K. Machemer, E. L. Braun, and E. Grotewold, 2011 Evolutionary and comparative analysis of MYB and bHLH plant transcription factors. Plant J 66: 94-116.

Han, M. V., G. W. C. Thomas, J. Lugo-Martinez, and M. W. Hahn, 2013 Estimating gene gain and loss rates in the presence of error in genome assembly and annotation using CAFE 3. Molecular Biology and Evolution 30(8): 1987-1997.

Jurka J.; V. V. Kapitonov, A. Pavlicek, P. Klonowski, O. Kohany, and J. Walichiewicz, 2005 Repbase Update, a database of eukaryotic repetitive elements. Cytogenet Genome Res 110: 462-467.

Kohany, O., A. J. Gentles, L. Hankus, J. Jurka, 2006 Annotation, submission and screening of repetitive elements in Repbase: RepbaseSubmitter and Censor. BMC Bioinformatics 7: 474.

Korf, I., 2004 Gene finding in novel genomes. BMC Bioinformatics 5: 59.

Liu, J., A. Osbourn, and P. Ma, 2015 MYB transcription factors as regulators of phenylpropanoid metabolism in plants. Mol. Plant 8: 689-708.

Parra G., K. Bradnam, and I. Korf, 2007 CEGMA: a pipeline to accurately annotate core genes in eukaryotic genomes. Bioinformatics 23: 1061-1067.

Price, A. L.; N. C. Jones, P. A. Pevzner, 2005 *De novo* identification of repeat families in large genomes. Bioinformatics 21: 351-358.

Simão, F. A., R. M. Waterhouse, P. Ioannidis, E. V. Kriventseva, and E. M. Zdobnov, 2015 BUSCO: assessing genome assembly and annotation completeness with single-copy orthologs. Bioinformatics 31(19): 3210-3212.

Slater, G.S. and E. Birney, 2005 Automated generation of heuristics for biological sequence comparison. BMC Bioinformatics 6: 31.

Stanke, M., M. Diekhans, R. Baertsch, and D. Haussler, 2008 Using native and syntenically mapped cDNA alignments to improve *de novo* gene finding. Bioinformatics, 24: 637-644.

Szklarczyk, D., A. Franceschini, S. Wyder, K. Forslund, D. Heller, *et al.*, 2015 STRING v10: protein–protein interaction networks, integrated over the tree of life. Nucleic Acids Research 43(D1): D447-D452.

Van Dongen, S., 2000 Graph clustering by flow simulation. PhD Thesis, University of Utrecht, The Netherlands.

Wu, T. D. and C. K. Watanabe, 2005 GMAP: A genomic mapping and alignment program for mRNA and EST sequences. Bioinformatics 21: 1859-1875.

Wicker, T.; F. Sabot, A. Hua-Van, J. L. Bennetzen, P. Capy, *et al.,* 2007 A unified classification system for eukaryotic transposable elements. Nature Reviews Genetics 8: 973-982.

Supplemental tables

Table S1. Raw sequence coverage for paired-end libraries. All libraries were sequenced to 151+151 bp on the HiSeq 2500 platform.

Library	Insert size (bp)	Read-pairs sequenced (millions)	Raw coverage (Mbp)
MGP_44_1_DF	710	128	38,574
MGP_44_2_DF	690	136	40,922
MGP_44_3_DF	662	133	40,290
MGP_44_4_DF	637	137	41,492
MGP_44_5_DF	613	135	40,678
MGP_44_6_DF	589	144	43,600
MGP_44_7_DF	568	143	43,306
MGP_44_8_DF	545	146	44,192
MGP_44_9_DF	521	140	42,414
MGP_44_10_DF	501	149	45,030
MGP_44_11_DF	480	144	43,500
MGP_44_12_DF	462	151	45,584
MGP_44_13_DF	444	141	42,644
MGP_44_14_DF	427	135	40,654
MGP_44_15_DF	409	139	41,996
MGP_44_16_DF	391	141	42,494
MGP_44_17_DF	372	142	42,758
MGP_44_18_DF	355	146	44,082
MGP_44_19_DF	338	145	43,914
MGP_44_20_DF	323	148	44,650
MGP_44_21_DF	308	145	43,646
MGP_44_22_DF	295	142	42,754
MGP_44_23_DF	283	148	44,570
MGP_44_24_DF	270	152	46,012
MGP_44_25_DF	238	144	43,462
MGP_44_26_DF	256	152	46,002
**Total**		**3,206**	**1,119,220**

Table S2. Raw sequence coverage for mate-pair libraries. All libraries were sequenced to 151+151 bp on the HiSeq 2500 platform.

Library	Insert size (bp)	Read-pairs sequenced (millions)	Raw coverage (Mbp)
DFMP_1	3,576	185	55,932
DFMP_2	5,864	115	34,734
DFMP_3	9,878	16	4,858
DFMP_4	3,515	252	76,194
DFMP_5	5,897	143	43,146
DFMP_6	9,965	11	3,442
DFMP_11	5,356	131	39,696
DFMP_12	6,234	117	35,248
DFMP_13	7,100	74	22,248
DFMP_14	8,990	4	1,080
DFMP_15	9,865	71	21,340
DFMP_16	11,454	31	9,442
DFMP_17	13,214	15	4,438
DFMP_18	15,486	13	4,072
DFMP_19	19,000	2	516
DFMP_20	20,756	1	402
DFMP_21	23,943	1	228
**Total**		**1,182**	**357,016**

Table S3. Exon and intron statistics for Douglas-fir gene models. Counts are provided for all four categories of classified models based on quality and completeness.

**A Intron statistics**						
**Gene model**	**Introns**	**Introns/ gene**	**Max. introns/ gene**	**Avg. size (bp)**	**Max. intron size (bp)**	**Intronic size (Mbp)**
High quality	145,595	425	68	2,301	182,831	335
High quality full-length	81,477	395	48	2,566	182,831	209
High quality partial	64,118	471	68	1,964	168,458	126
Low quality	38,526	283	36	2,704	269,670	104
**B Exon statistics**						
**Gene model**	**Exons**			**Avg. size (bp)**	**Max. exon size (bp)**	**Exonic size (Mbp)**
High quality	181,475			230	8,036	42
High quality full-length	103,734			247	8,036	26
High quality partial	77,741			208	8,012	16
Low quality	57,476			221	5,778	13

Table S4. Intron-exon splice junction statistics.

Splice donor site (5' end)	Percentage	Splice acceptor site (3' end)	Percentage
AA	0.002	**AA**	**0.050**
AC	0.002	**AC**	**0.033**
**GT**	**99.83**	GT	0.002
NN	0.001	NN	0.001
AG	0.003	**AG**	**99.83**
CC	0.002	CC	0.001
TT	0.018	TT	0.002
GG	0.007	CG	0.003
**GC**	**0.054**	TC	0.002
**AT**	**0.050**	GG	0.010
GA	0.012	GC	0.002
GN	0.001	AT	0.045
TG	0.001	GA	0.002
TA	0.002	TG	0.017
CA	0.001	CA	0.002
TC	0.002	CT	0.001

Table S5. Rates of gene-family evolution examined across 16 land plants **[separate Excel file]**

Table S6. Summary of gene-family evolution examined across land plants and among the sequenced species of Pinaceae

	λ without error	λ with error	λ with species-specific error
**Land plants**			
Dicots	0.00252	0.00218	0.00180
Monocots	0.00208	0.00189	0.00203
Pinaceae	0.00340	0.00336	0.00338
Other branches	0.00040	0.00040	0.00041
**Pinaceae**			
Pine trees	0.0073	0.0073	
Norway spruce	0.0036	0.0036	
Douglas-fir	0.0035	0.0035	

Table S7. Lineage-specific gene turnover error rates estimated by CAFÉ. Species codes as in Figure 3.

	Species	Error rate	Average error rate
Dicots	Athaliana	0	0.1640625
	Ptrichocarpa	0.253125	
	Tcacao	0	
	Rcommunis	0.3515625	
	Mesculenta	0.3796875	
	Ppersica	0	
Monocots	Osativa	0.0140625	0.01828125
	Bdistachyon	0	
	Sbicolor	0.028125	
	Sitalica	0.02109375	
	Macuminata	0.028125	
Pinaceae	Pila	0.30234375	0.181054688
	Psme	0.084375	
	Pita	0.1125	
	Pabies	0.225	
Bryophyte	Ppatens	0.028125	

Table S8. Summary of gene-family evolution and lineage-specific expansions across land plants **[separate Excel file]**

Table S9. Genetic networks identified with significant gene losses in Douglas-fir **[separate Excel file]**

Table S10. Genetic networks identified with significant gene duplications in Douglas-fir **[separate Excel file]**

Table S11. Summary of gene-family evolution examined within Pinaceae **[separate Excel file]**

Table S12. Summary of gene-family evolution and lineage-specific expansions within Pinaceae **[separate Excel file]**

Table S13. Protein angiosperm database compiled from PLAZA. Total protein sequences are reported for the selected gymnosperms and angiosperms. These sequences were used as evidence for annotation with MAKER-P.

		Species	Number of proteins reported
Gymnosperm		*Picea abies*	25,974
		Picea glauca	13,026
		Pinus taeda	8,901
		Pinus lambertiana	13,936
	**Total**		**61,837**
Angiosperm		*Eucalyptus grandis*	36,449
		Oryza sativa	40,709
		Vitis vinifera	26,182
		Populus trichocarpa	41,434
		Amborella trichopoda	26,460
		Physcomitrella patens	32,390
	**Total**		**203,624**

Table S14. Resources for MAKER-P annotation sourced from Expressed Sequence Tags (ESTs) from the *de novo* assembled needle transcriptome of Douglas-fir

Source	Species	Number of ESTs
Needle transcriptome (frame selected)	*Pseudotsuga menziesii*	65,102
GenBank sourced (accessed November 2015)	*Pseudotsuga menziesii*	20,583
**Total**		**85,685**

Table S15. Resources for MAKER-P annotation sourced from Expressed Sequence Tags (ESTs) and *de novo* assembled transcriptomes of conifer species

Source	Taxon	Number of ESTs
De novo assembled transcriptomes	*Pinus lambertiana*	42,475
	Pinus monticola	10,494
	Pinus albicaulis	23,862
	Pinus flexilis	14,238
	Pinus taeda	21,346
	Picea sitchensis	4,631
GenBank sourced (accessed December 2015)	*Pinus* genus	477,316
	Picea genus	543,623
	Cryptomeria genus	61,500
**Total**		**1,119,485**

Table S16. Transposable elements contributing to 20% of the Douglas-fir repeat content

Repeat element	Family	Frequency	Total length (bp)	Genome (%)
rnd-4_family-22	LTR/Copia	112,382	2.55E+08	1.80
rnd-5_family-107	LTR/Gypsy	60,173	2.29E+08	1.62
rnd-3_family-235	LTR/Gypsy	111,252	2.26E+08	1.59
rnd-4_family-682	LTR/Copia	60,373	2.14E+08	1.51
rnd-5_family-283	LTR	109,089	1.82E+08	1.28
rnd-6_family-376	LTR	124,590	1.81E+08	1.28
rnd-2_family-5	LTR/Gypsy	40,660	1.71E+08	1.20
rnd-3_family-168	LTR/Gypsy	33,869	1.60E+08	1.12
rnd-5_family-517	LTR/Gypsy	64,553	1.26E+08	0.89
rnd-6_family-658	LTR/Gypsy	87,889	1.21E+08	0.86
rnd-6_family-1236	LTR/Gypsy	61,092	1.16E+08	0.82
rnd-2_family-58	LTR/Gypsy	87,599	1.09E+08	0.77
rnd-4_family-133	LTR/Copia	96,558	1.06E+08	0.74
rnd-6_family-1393	LTR	47,592	1.04E+08	0.73
rnd-3_family-395	LTR/Gypsy	45,262	1.02E+08	0.72
rnd-3_family-36	LTR	98,056	1.00E+08	0.70
rnd-5_family-109	LTR/Gypsy	46,904	97,969,430	0.69
rnd-5_family-26	LTR/Gypsy	86,765	95,074,635	0.67
rnd-4_family-308	LTR/Gypsy	60,131	92,762,481	0.65
rnd-6_family-224	LTR	52,587	91,306,488	0.64
**Total**				**20.28**

Table S17. Summary of predicted secretory motifs in the Douglas-fir gene models

Prediction method	Total proteins
Total number of genome-predicted proteins (HQ)	22,257
ChloroP signal	1,681
SignalP signal	1,540
TargetP signal	3,547
Phobius signal	1,734
TMHMM	19,314
ER signal	13
Core secretory proteins	895

Supplemental figure legends

Figure S1. The histogram of 24-mer depth for our target megagametophyte. The 'haploid' single-copy peak has the expected depth of 52X. There are 27E10 total 24-mers comprising the single-copy peak, 6.1E10 24-mers at twice single-copy depth, and 2.7E10 24-mers at three times single-copy depth. These observations are consistent with sequencing a haploid genome comprised mainly of ancient (diverged) copies of transposable elements.

FigureS2. Protein alignment conservation of the PAL genes across major land plant groups including conifers and ancestral Gnetales (only N-terminal region shown). The conserved motif MIO region (GTITASGDLVPLSYIAG; Ala-Ser-Gly triad) is highlighted as a visual reference.

Figure S3. Gene duplications and gene losses in gene families with significant high turnover rates. The red, blue, and green branches correspond to dicots, monocots, and Pinaceae, respectively. Numbers separated by a slash on or nearby each branch indicate gene duplications (left of slash) and gene losses (right of slash). The scale bar is in million years. Species codes as in Figure 3.

Figure S4. Phylogenetic tree comparing Douglas-fir and other conifers together with select angiosperm light-harvesting-complex proteins. Members of the genera *Pinus* (pink) and *Picea* (blue) are shown in the two innermost data bars circling the labels. Antenna proteins of PSII are LHCb1 (green clade) and LHCb2 (cyan clade), LHCb3 (purple clade), LHCb4 (navy blue clade), LHCb5 (orange clade), and LHCb6 (red clade). Antenna proteins of PSI are shown in clades with dashed lines. The clade harboring LHCa5 proteins is shown in green dashed lines. The tree is rooted using the *Chlamydomonas* LHCbm1 sequence as an outgroup. Proteins carrying WYGPDR-trimerization domains are indicated with the purple data bar. A fraction of the LHCb1 proteins carrying WYGKDR domains is shown with a pale purple databar. Proteins carrying other motifs WYG[PER/SDR/PSR/QDR/ADR/PNR/PDW/PDV/RWL], FYG[PER/PDR/PNR], and WYXPDR are shown with another purple bar in the outermost circle. An interactive version of this figure is available at http://itol.embl.de/tree/1379989172344871490022208# and excerpted details are given in Figure 4.

Figure S5. Conifer clades of D1/D2 proteins rooted using the earliest forms of D1/D2 reaction-center proteins from *Gloeobacter kilaueensis* (red clades) which possesses four D1 paralogs that functionally form a gradient from anoxic to oxygen-evolving reaction centers. D1 (green) and D2 (orange) form two distinct clades. *Arabidopsis* orthologs (red labels) are nested in each clade. There appear to be at least three D1 protein paralogs represented in Douglas-fir transcriptomes (PSME and IOVS-1KP).

Figure S6. A phylogenetic tree comparing Douglas-fir (bold labels) and other conifers together with select angiosperm red/far-red-light-sensing phytochrome photoreceptor proteins. The tree has been rooted using *Physcomitrella patens*. The green clade is PhyP, the orange is PhyO, the navy is PhyN, and the green is photolyase-domain-containing cryptochrome. Databars represent shade-tolerance categories: tolerant (blue), intolerant (red), and intermediate tolerant (green). Four-letter species codes and sequence IDs from 1KP database are included in the labels.

Figure S7. A phylogenetic tree comparing Douglas-fir (bold labels) and other conifers together with select angiosperm blue-light-sensing cryptochrome photoreceptor proteins. The tree has been rooted using *Physcomitrella patens*. The cyan clade is CRY1, the orange is CRY2, the navy is CRY3, and the green is photolyase-domain-containing cryptochrome. Databars represent shade-tolerance categories: tolerant (blue), intolerant (red), and intermediate tolerant (green). Four-letter species codes and sequence IDs from 1KP database are included in the labels.

Figure S8. A phylogenetic tree comparing Douglas-fir (bold labels) and other conifers together with select angiosperm UV/blue-light-sensing phototropin proteins. The tree has been rooted using *Physcomitrella patens*. The shade-intolerant pine genus is shown in the blue clade. Databars represent shade-tolerance categories: tolerant (blue), intolerant (red), and intermediate tolerant (green). Four-letter species codes and sequence IDs from 1KP database are included in the labels.

Figure S9. A phylogenetic tree comparing Douglas-fir (bold labels) and other conifers together with select angiosperm PsBs proteins. The tree has been rooted using *Chlamydomonas*. Databars represent shade-tolerance

categories: tolerant (blue), intolerant (red). Transcriptomic data from this study are labeled RA. Four-letter species codes and sequence IDs from 1KP database are included in the labels.

Figure S10. A phylogenetic tree comparing Douglas-fir (bold labels) and other conifers together with select angiosperm VDE enzymes. The tree has been rooted using *Chlamydomonas*. Databars represent shade-tolerance categories: tolerant (blue), intolerant (red). Transcriptomic data from this study are labeled RA. Four-letter species codes and sequence IDs from 1KP database are included in the labels.

Figure S11. A phylogenetic tree comparing Douglas-fir (bold labels) and other conifers together with select angiosperm PSAH1 proteins. The tree has been rooted using *Chlamydomonas*. Databars represent shade-tolerance categories: tolerant (blue), intolerant (red). Transcriptomic data from this study are labeled PSME. Four-letter species codes and sequence IDs from 1KP database are included in the labels.

Figure S12. A phylogenetic tree comparing Douglas-fir (bold labels) and other conifers together with select angiosperm STN7 proteins. The tree has been rooted using *Chlamydomonas*. Members of basal gymnosperms *Welwitschia*, *Gnetum*, and *Ephedra* are shown in navy blue clades. Angiosperms including basal members such as *Amborella* and *Nelumbo* are shown in the cyan clade. Databars represent shade-tolerance categories: tolerant (blue), intolerant (red). Transcriptomic data from this study are labeled RA. Four-letter species codes and sequence IDs from the 1KP database are included in the labels.

Figure S13. (**A**) Genome sizes of three sequenced conifers: *Pseudotsuga menziesii*, *Pinus lambertiana*, and *Pinus taeda* and their respective proportions of DNA and retrotransposons in intergenic (windows size=150 kbp) and intronic positions. (**B**) Boxplot of intron lengths in *Pinus lambertiana*, *Pinus taeda*, and *Pseudotsuga menziesii*. (**C**) Boxplot of coding-sequence lengths in *Pinus lambertiana*, *Pinus taeda*, and *Pseudotsuga menziesii*.

Figure S14. Proportional distribution of classified interspersed repeats in the Douglas-fir genome.

Figure S15. (**A**) Prediction and distribution of the secretory proteins (core set of 895 secretory proteins). (**B**) Distribution of transcription factors (TF) highlighting dominance of the MYB family.
