# Supplementary material for: The Douglas-Fir Genome Sequence Reveals Specialization of the Photosynthetic Apparatus in Pinaceae
Source: G3 (Bethesda). 2017 Jul 27;7(9):3157–67. doi: 10.1534/g3.117.300078 (PMC5592940; doi:10.1534/g3.117.300078)
Supplement: Supplementary file 2 [file 3157S2.pdf]

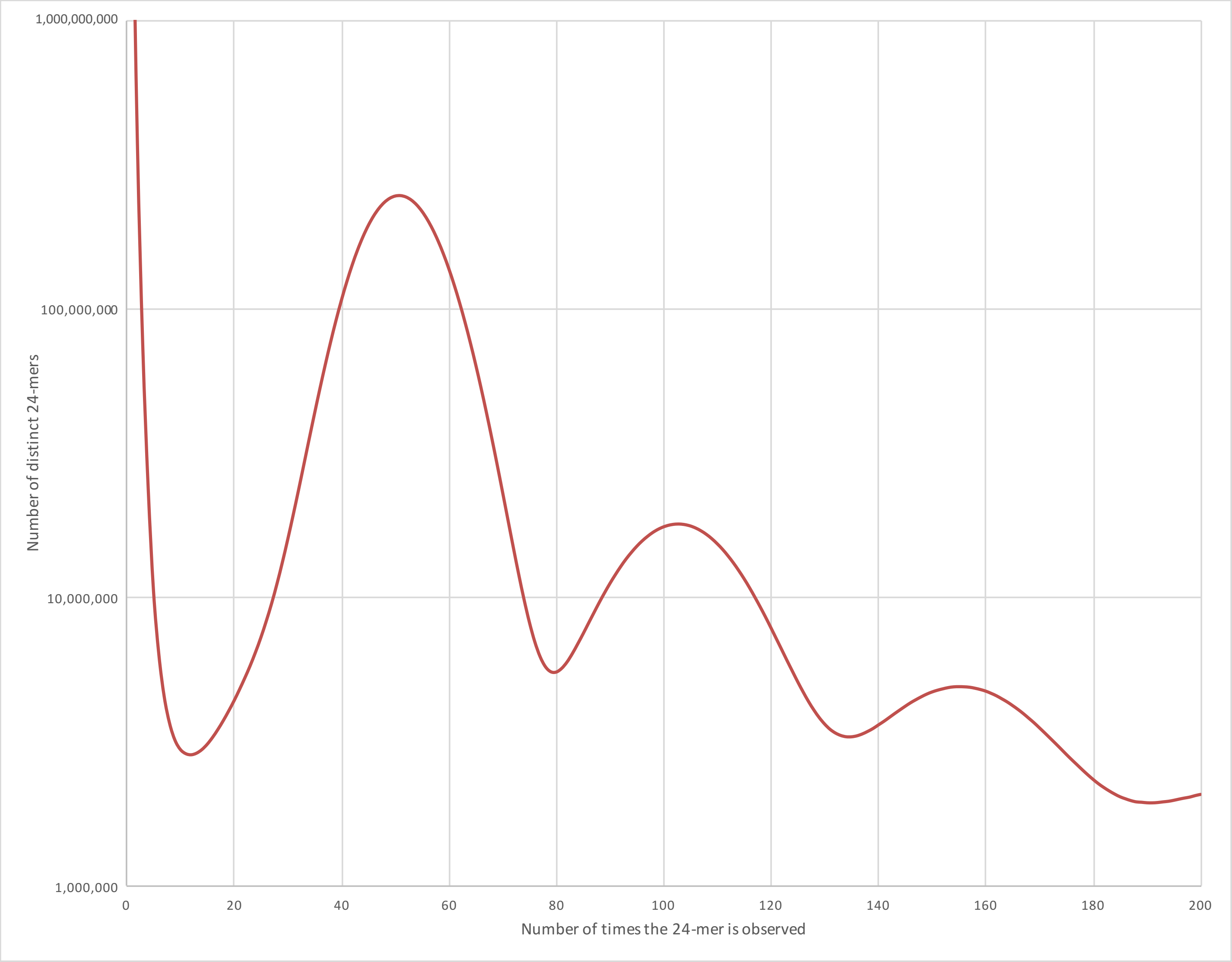

Figure S1. The histogram of 24-mer depth for our target megagametophyte. The 'haploid' single-copy peak has the expected depth of 52X. There are 27E10 total 24-mers comprising the single-copy peak, 6.1E10 24-mers at twice single-copy depth, and 2.7E10 24-mers at three times single-copy depth. These observations are consistent with sequencing a haploid genome comprised mainly of ancient (diverged) copies of transposable elements.

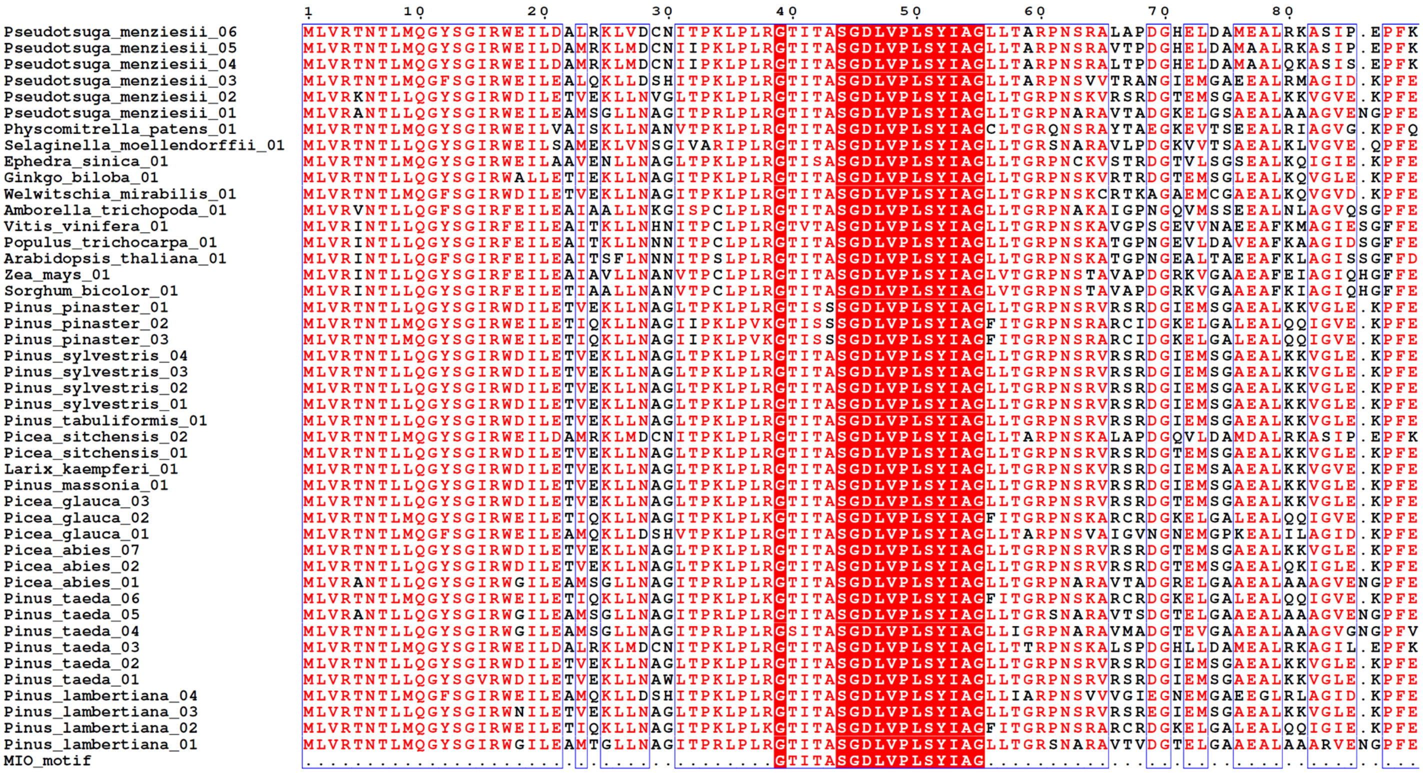

Figure S2. Protein alignment conservation of the PAL genes across major land plant groups including conifers and ancestral Gnetales (only N-terminal region shown). The conserved motif MIO region (GTITAS-GDLVPLSYIAG; Ala-Ser-Gly triad) is highlighted as a visual reference.

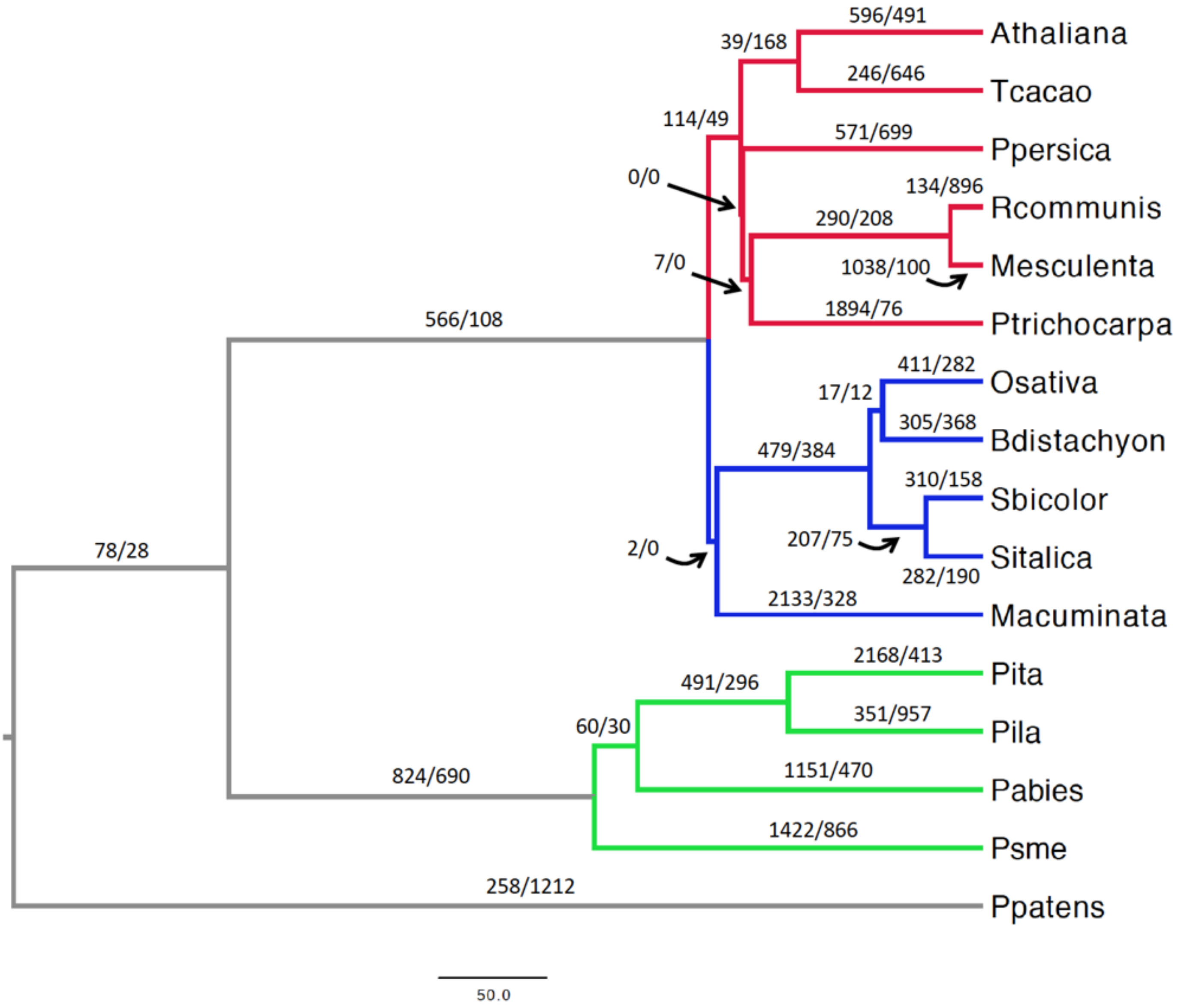

Figure S3. Gene duplications and gene losses in gene families with significant high turnover rates. The red, blue, and green branches correspond to dicots, monocots, and Pinaceae, respectively. Numbers separated by a slash on or nearby each branch indicate gene duplications (left of slash) and gene losses (right of slash). The scale bar is in million years. Species codes as in Figure 3.

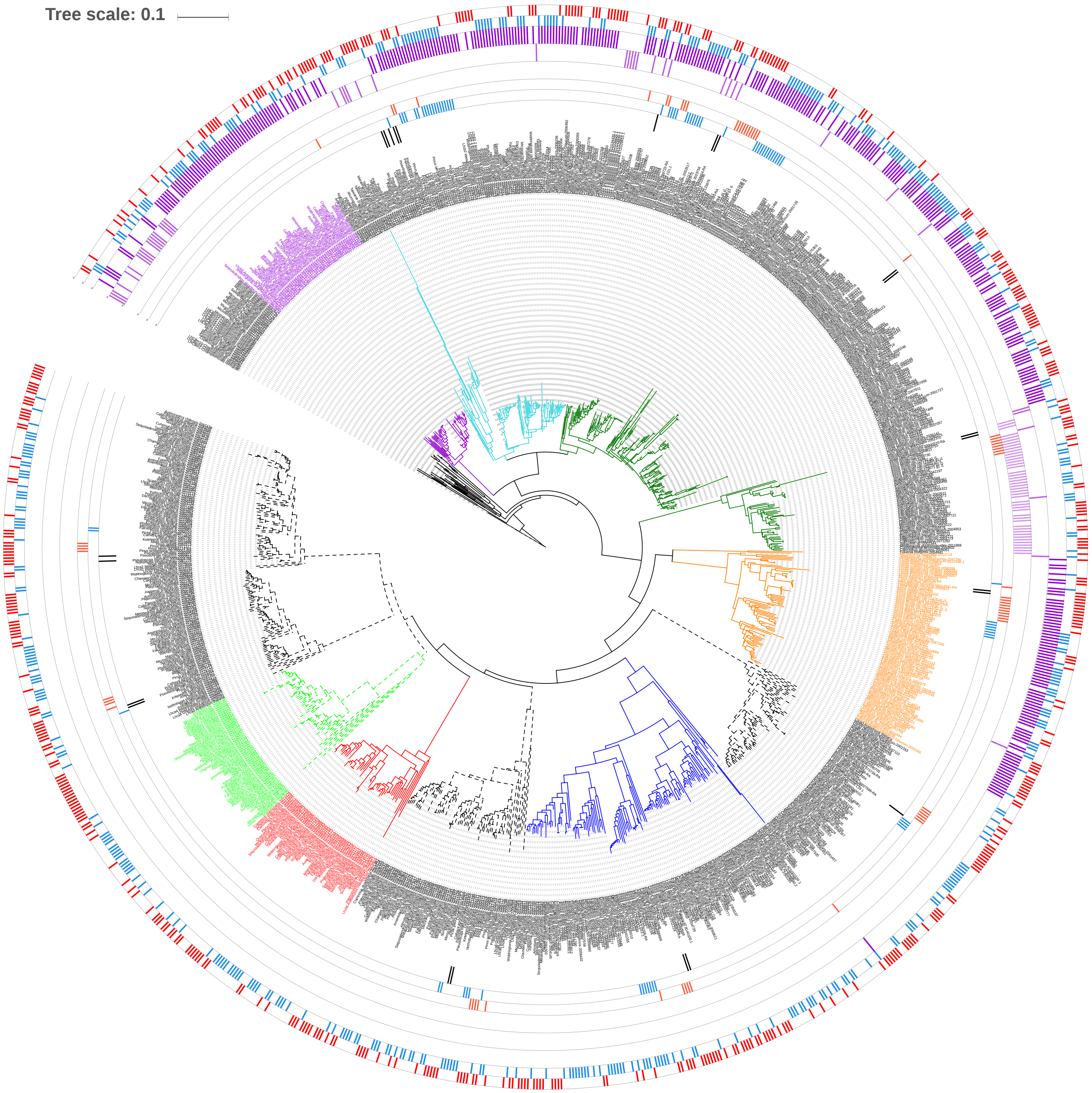

Figure S4. Phylogenetic tree comparing Douglas-fir and other conifers together with select angiosperm light-harvesting-complex proteins. Members of the genera *Pinus* (pink) and *Picea* (blue) are shown in the two innermost data bars circling the labels. Antenna proteins of PSII are LHCb1 (green clade) and LHCb2 (cyan clade), LHCb3 (purple clade), LHCb4 (navy blue clade), LHCb5 (orange clade), and LHCb6 (red clade). Antenna proteins of PSI are shown in clades with dashed lines. The clade harboring LHCa5 proteins is shown in green dashed lines. The tree is rooted using the *Chlamydomonas* LHCbm1 sequence as an outgroup. Proteins carrying WYGPDR-trimerization domains are indicated with the purple data bar. A fraction of the LHCb1 proteins carrying WYGKDR domains is shown with a pale purple databar. Proteins carrying other motifs WYG[PER/SDR/PSR/QDR/ADR/ PNR/PDW/PDV/RWL], FYG[PER/PDR/PNR], and WYXPDR are shown with another purple bar in the outermost circle. An interactive version of this figure is available at http://itol.embl.de/tree/1379989172344871490022208# and excerpted details are given in Figure 4.

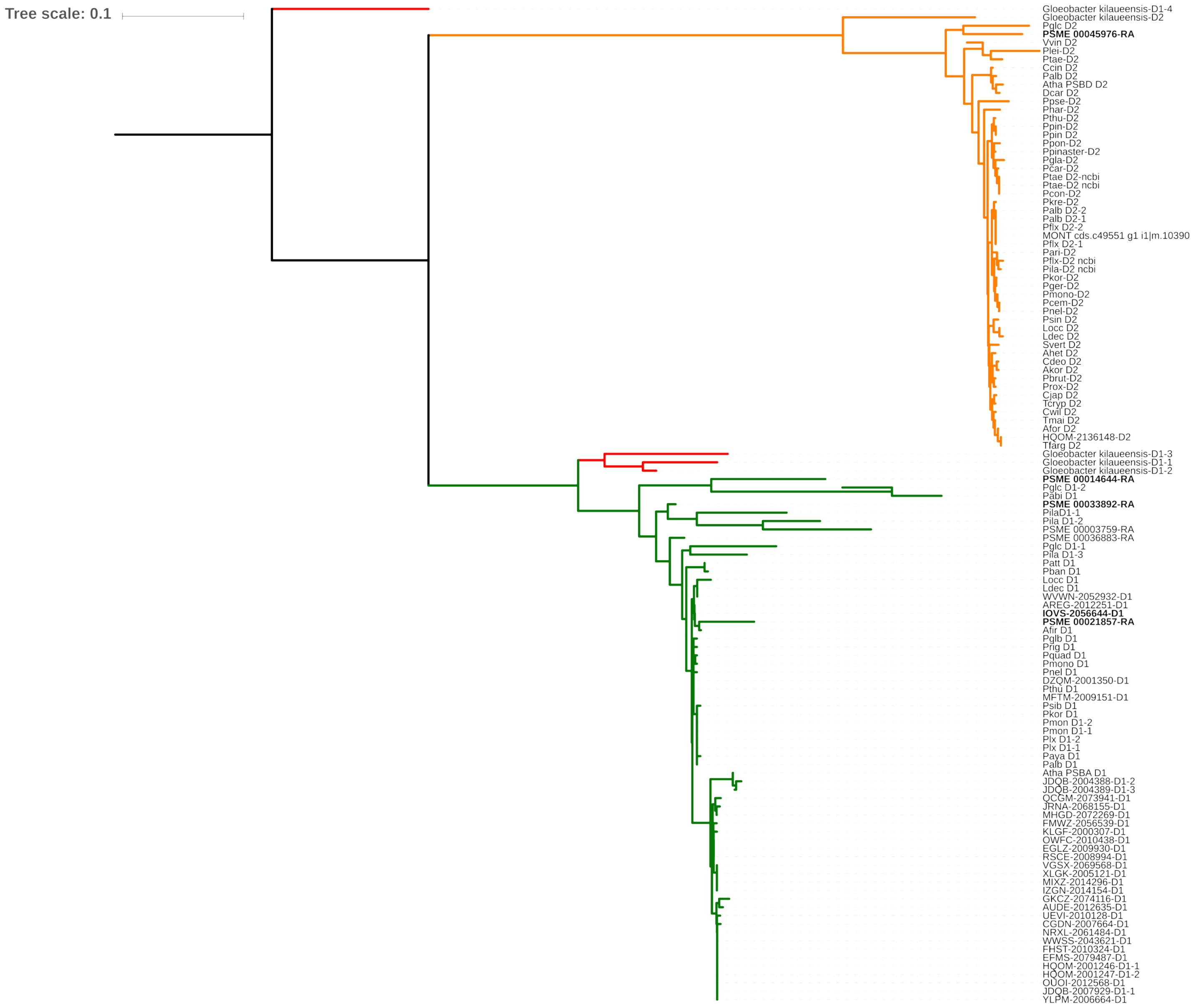

Figure S5. Conifer clades of D1/D2 proteins rooted using the earliest forms of D1/D2 reaction-center proteins from *Gloeobacter kilaueensis* (red clades) which possesses four D1 paralogs that functionally form a gradient from anoxic to oxygen-evolving reaction centers. D1 (green) and D2 (orange) form two distinct clades. *Arabidopsis* orthologs (red labels) are nested in each clade. There appear to be at least three D1 protein paralogs represented in Douglas-fir transcriptomes (PSME and IOVS-1KP).

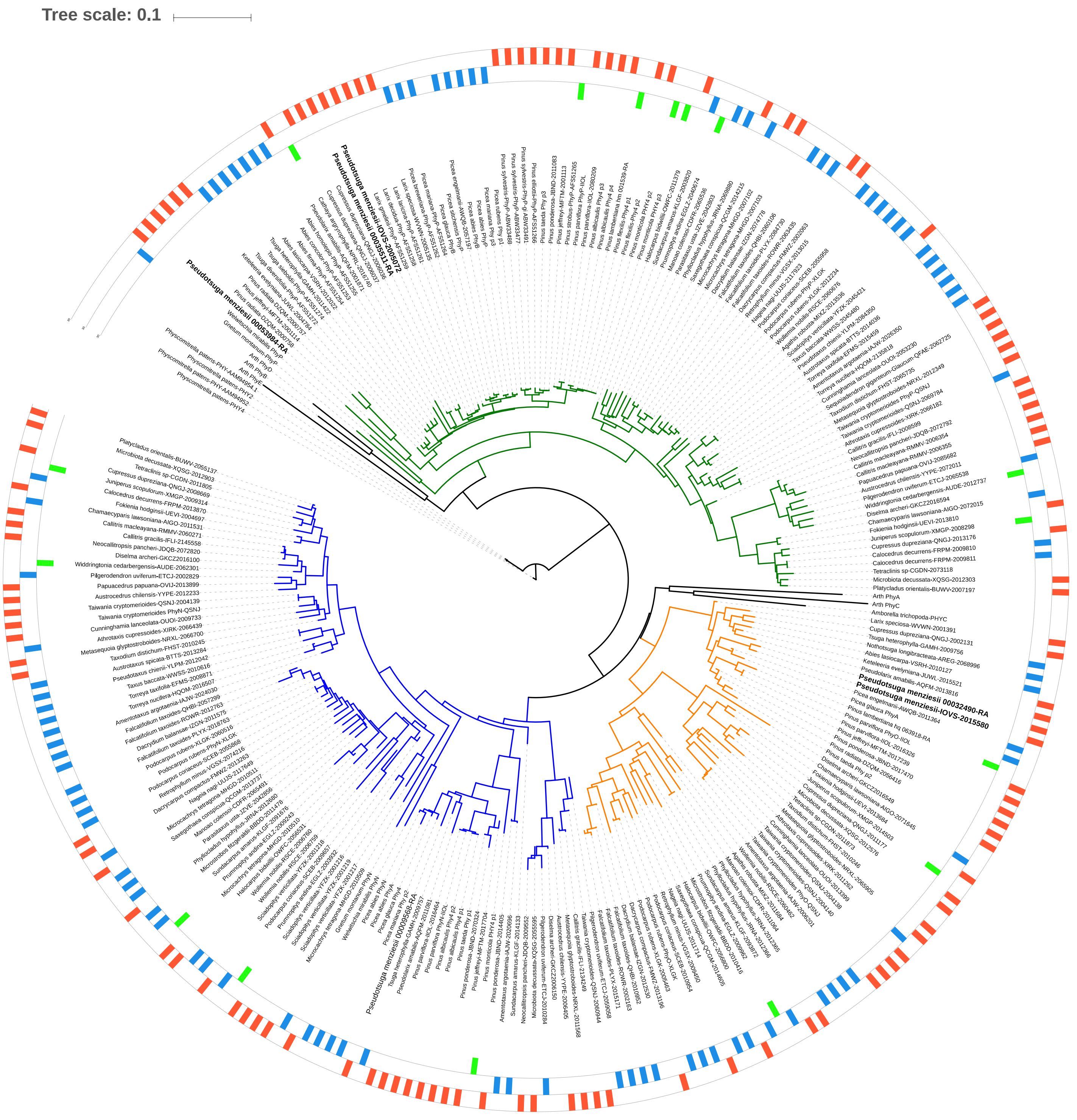

Figure S6. A phylogenetic tree comparing Douglas-fir (bold labels) and other conifers together with select angiosperm red/far-red-light-sensing phytochrome photoreceptor proteins. The tree has been rooted using *Physcomitrella patens*. The green clade is PhyP, the orange is PhyO, the navy is PhyN, and the green is photolyase-domain-containing cryptochrome. Databars represent shade-tolerance categories: tolerant (blue), intolerant (red), and intermediate tolerant (green). Four-letter species codes and sequence IDs from 1KP database are included in the labels.

Reference genome of Douglas-fir

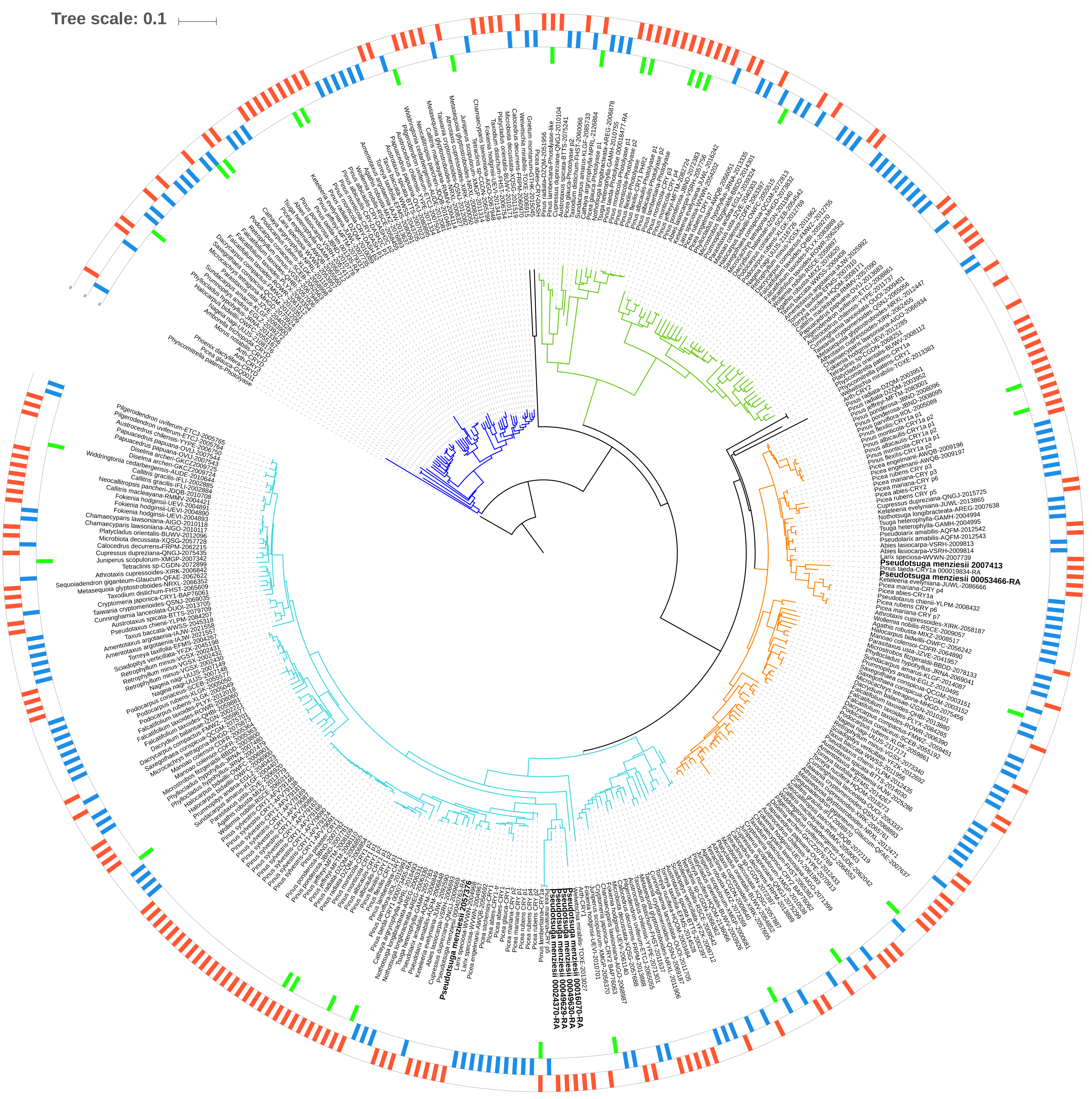

Figure S7. A phylogenetic tree comparing Douglas-fir (bold labels) and other conifers together with select angiosperm blue-light-sensing cryptochrome photoreceptor proteins. The tree has been rooted using *Physcomitrella patens*. The cyan clade is CRY1, the orange is CRY2, the navy is CRY3, and the green is photolyase-domain-containing cryptochrome. Databars represent shade-tolerance categories: tolerant (blue), intolerant (red), and intermediate tolerant (green). Four-letter species codes and sequence IDs from 1KP database are included in the labels.

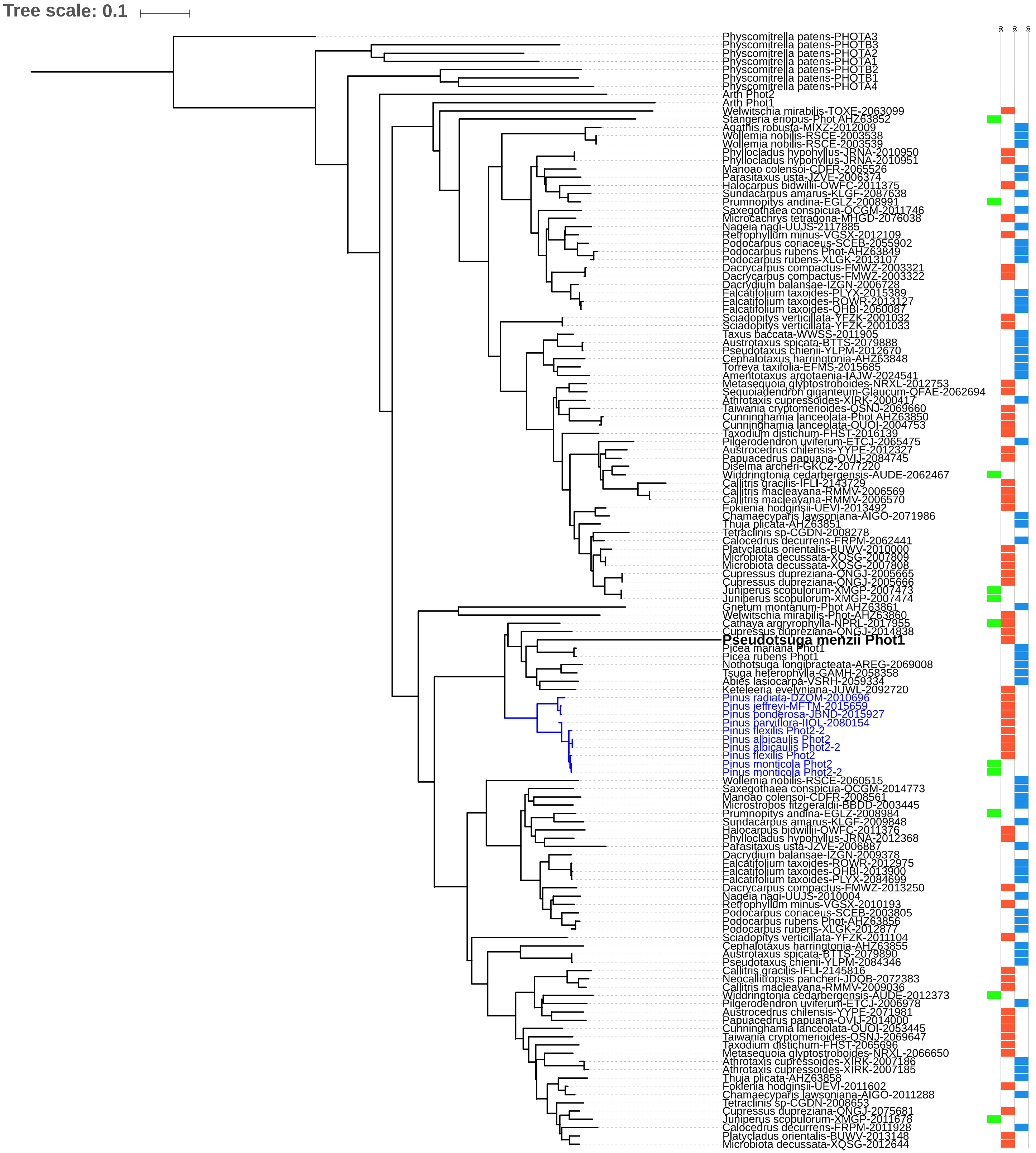

Figure S8. A phylogenetic tree comparing Douglas-fir (bold labels) and other conifers together with select angiosperm UV/blue-light-sensing phototropin proteins. The tree has been rooted using *Physcomitrella patens*. The shade-intolerant pine genus is shown in the blue clade. Databars represent shade-tolerance categories: tolerant (blue), intolerant (red), and intermediate tolerant (green). Four-letter species codes and sequence IDs from 1KP database are included in the labels..

Reference genome of Douglas-fir

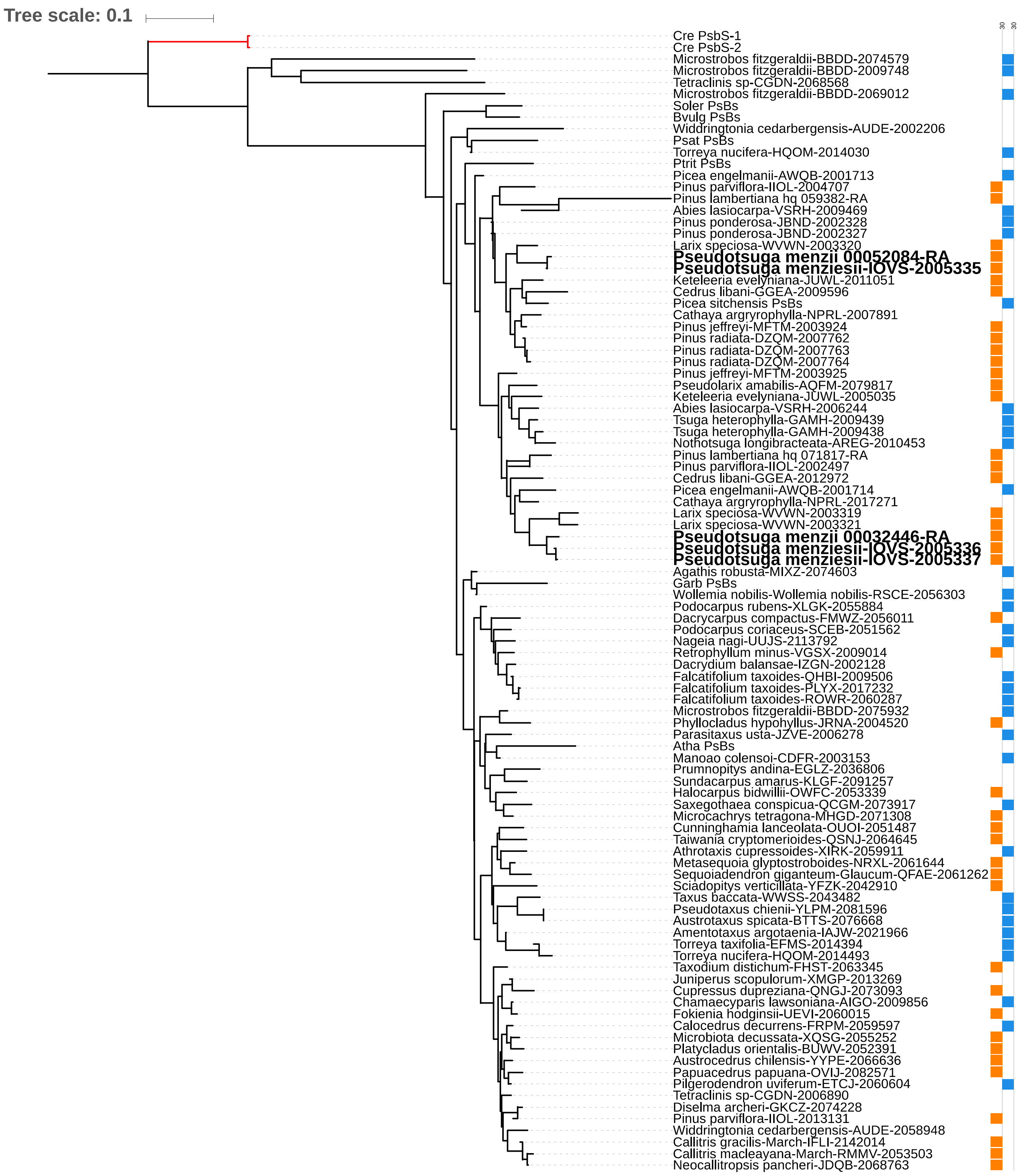

Figure S9. A phylogenetic tree comparing Douglas-fir (bold labels) and other conifers together with select angiosperm PsBs proteins. The tree has been rooted using *Chlamydomonas*. Databars represent shade-tolerance categories: tolerant (blue), intolerant (red). Transcriptomic data from this study are labeled RA. Four-letter species codes and sequence IDs from 1KP database are included in the labels.

Reference genome of Douglas-fir

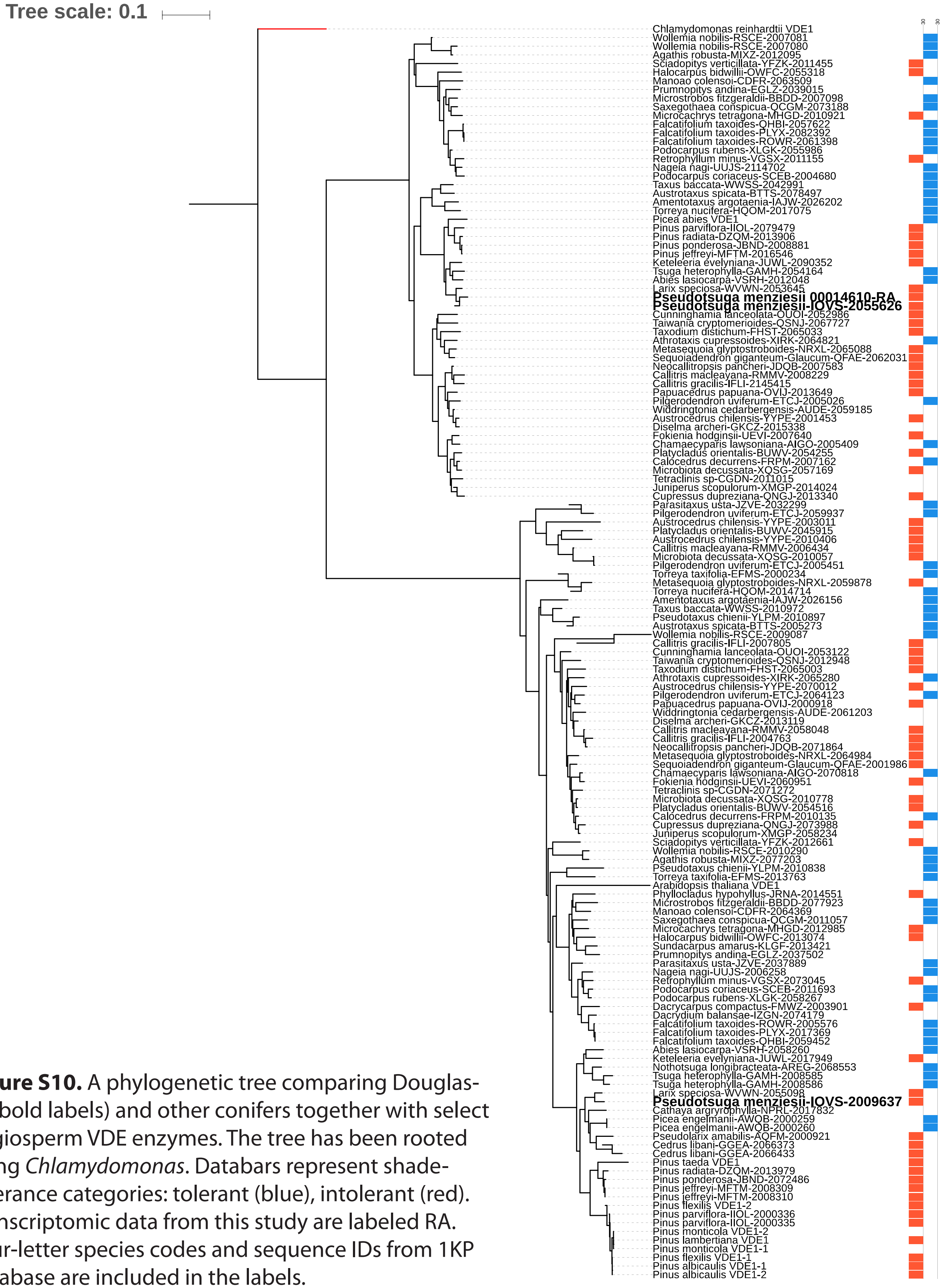

Figure S10. A phylogenetic tree comparing Douglas-fir (bold labels) and other conifers together with select angiosperm VDE enzymes. The tree has been rooted using *Chlamydomonas*. Databars represent shade-tolerance categories: tolerant (blue), intolerant (red). Transcriptomic data from this study are labeled RA. Four-letter species codes and sequence IDs from 1KP database are included in the labels.

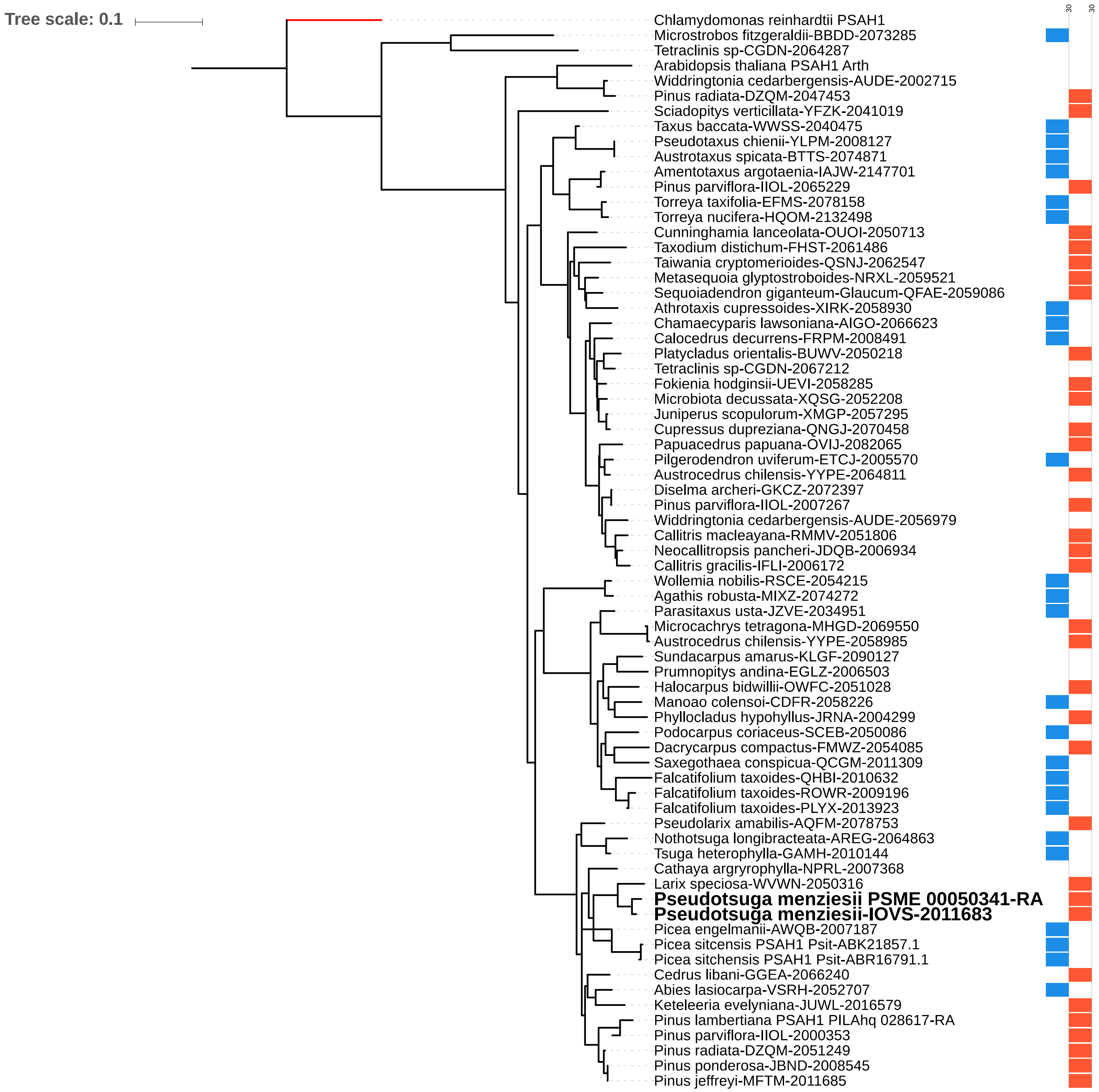

Figure S11. A phylogenetic tree comparing Douglas-fir (bold labels) and other conifers together with select angiosperm PSAH1 proteins. The tree has been rooted using *Chlamydomonas*. Databars represent shade-tolerance categories: tolerant (blue), intolerant (red). Transcriptomic data from this study are labeled PSME. Four-letter species codes and sequence IDs from 1KP database are included in the labels.

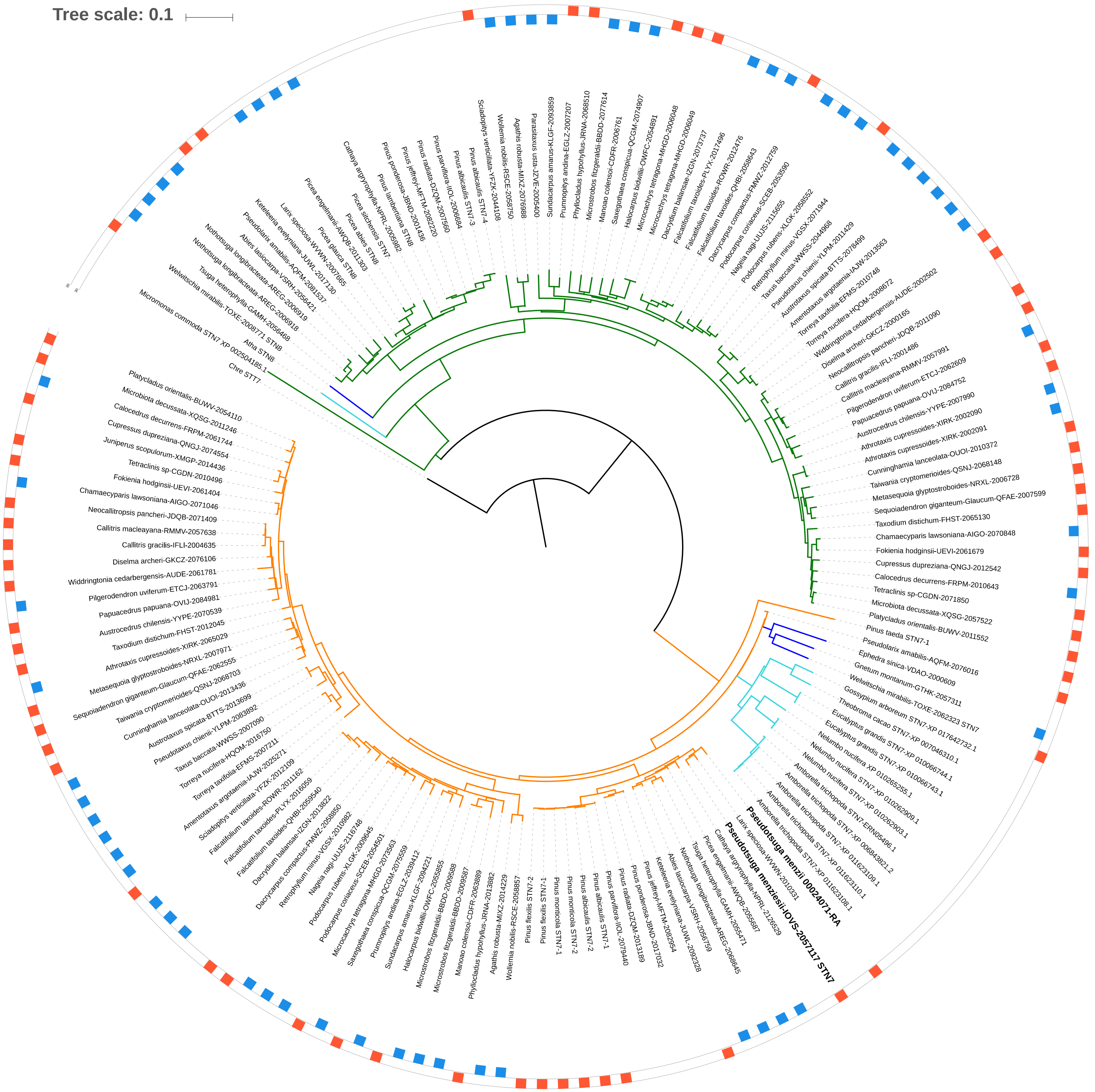

Figure S12. A phylogenetic tree comparing Douglas-fir (bold labels) and other conifers together with select angiosperm STN7 proteins. The tree has been rooted using *Chlamydomonas*. Members of basal gymnosperms *Welwitschia*, *Gnetum*, and *Ephedra* are shown in navy blue clades. Angiosperms including basal members such as *Amborella* and *Nelumbo* are shown in the cyan clade. Databars represent shade-tolerance categories: tolerant (blue), intolerant (red). Transcriptomic data from this study are labeled RA. Four-letter species codes and sequence IDs from the 1KP database are included in the labels.

Reference genome of Douglas-fir

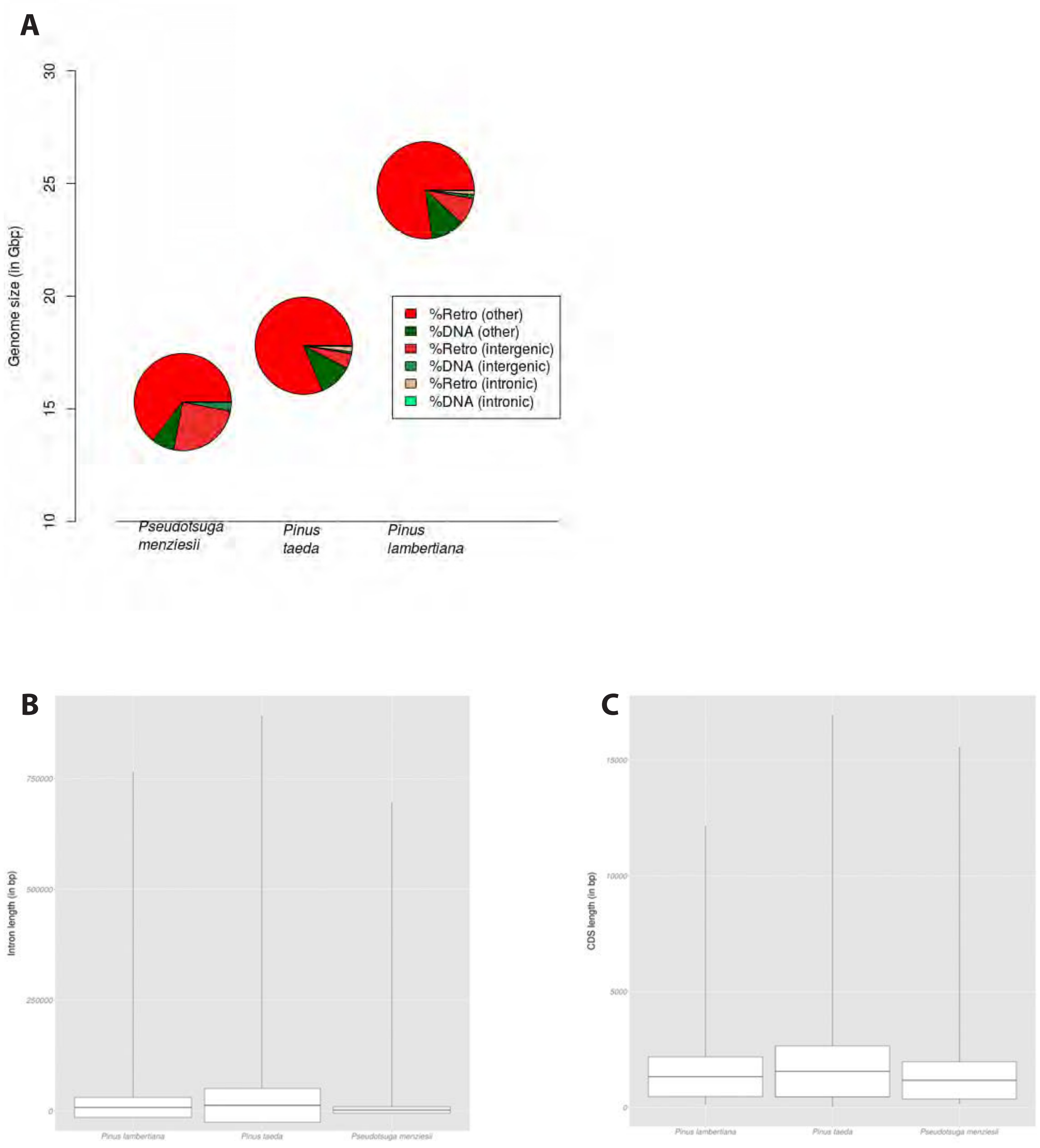

Figure S13. (**A**) Genome sizes of three sequenced conifers: *Pseudotsuga menziesii*, *Pinus lambertiana*, and *Pinus taeda* and their respective proportions of DNA and retrotransposons in intergenic (windows size=150 kbp) and intronic positions. (**B**) Boxplot of intron lengths in *Pinus lambertiana*, *Pinus taeda*, and *Pseudotsuga menziesii*. (**C**) Boxplot of coding-sequence lengths in *Pinus lambertiana*, *Pinus taeda*, and *Pseudotsuga menziesii*.

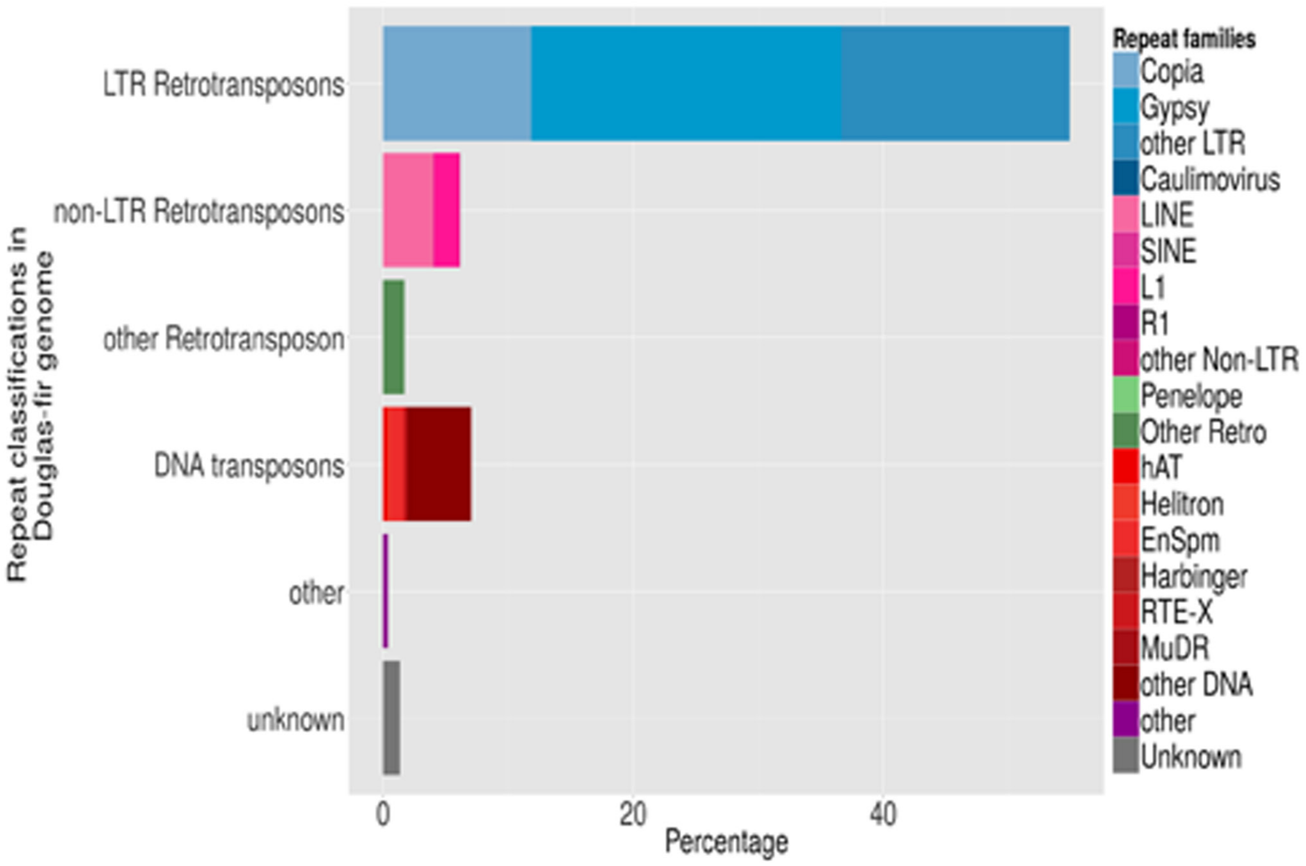

Figure S14. Proportional distribution of classified interspersed repeats in the Douglas-fir genome.

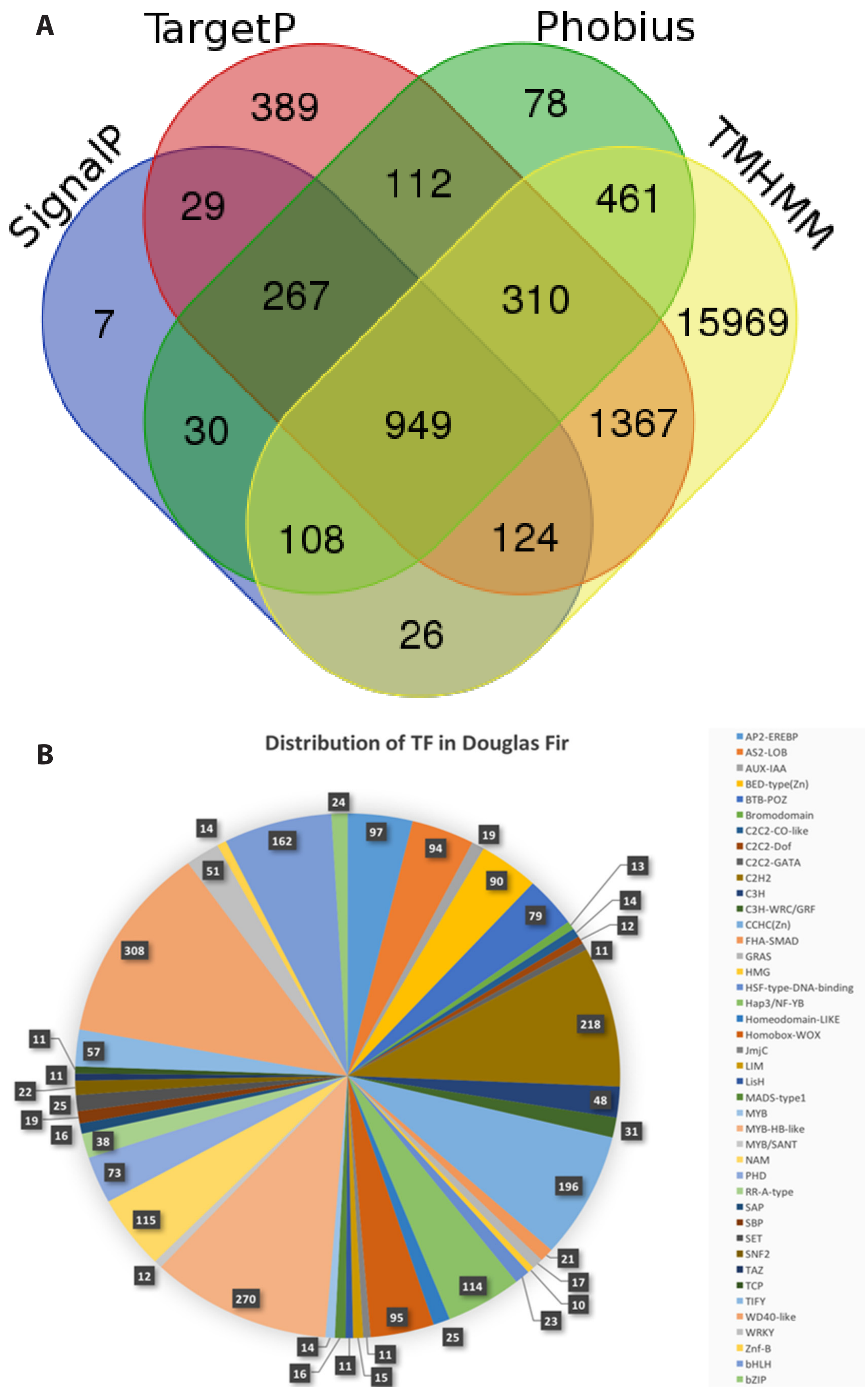

Figure S15. (**A**) Prediction and distribution of the secretory proteins (core set of 895 secretory proteins). (**B**) Distribution of transcription factors (TF) highlighting dominance of the MYB family.
